# Frequency‐specific coactivation patterns in resting‐state and their alterations in schizophrenia: An fMRI study

**DOI:** 10.1002/hbm.25884

**Published:** 2022-04-27

**Authors:** Hang Yang, Hong Zhang, Chun Meng, Afra Wohlschläger, Felix Brandl, Xin Di, Shuai Wang, Lin Tian, Bharat Biswal

**Affiliations:** ^1^ The Clinical Hospital of Chengdu Brain Science Institute, MOE Key Laboratory for Neuroinformation, Center for Information in Medicine, School of Life Science and Technology University of Electronic Science and Technology of China Chengdu China; ^2^ Department of Neuroradiology, TUM‐Neuroimaging Center Technical University of Munich (TUM) Munich Germany; ^3^ Department of Psychiatry, TUM‐Neuroimaging Center Technical University of Munich (TUM) Munich Germany; ^4^ Department of Biomedical Engineering New Jersey Institute of Technology Newark New Jersey USA; ^5^ Department of Psychiatry The Affiliated Wuxi Mental Health Center of Nanjing Medical University Wuxi China

**Keywords:** coactivation patterns, dynamics, frequency‐specific, schizophrenia

## Abstract

The resting‐state human brain is a dynamic system that shows frequency‐dependent characteristics. Recent studies demonstrate that coactivation pattern (CAP) analysis can identify recurring brain states with similar coactivation configurations. However, it is unclear whether and how CAPs depend on the frequency bands. The current study investigated the spatial and temporal characteristics of CAPs in the four frequency sub‐bands from slow‐5 (0.01–0.027 Hz), slow‐4 (0.027–0.073 Hz), slow‐3 (0.073–0.198 Hz), to slow‐2 (0.198–0.25 Hz), in addition to the typical low‐frequency range (0.01–0.08 Hz). In the healthy subjects, six CAP states were obtained at each frequency band in line with our prior study. Similar spatial patterns with the typical range were observed in slow‐5, 4, and 3, but not in slow‐2. While the frequency increased, all CAP states displayed shorter persistence, which caused more between‐state transitions. Specifically, from slow‐5 to slow‐4, the coactivation not only changed significantly in distributed cortical networks, but also increased in the basal ganglia as well as the amygdala. Schizophrenia patients showed significant alteration in the persistence of CAPs of slow‐5. Using leave‐one‐pair‐out, hold‐out and resampling validations, the highest classification accuracy (84%) was achieved by slow‐4 among different frequency bands. In conclusion, our findings provide novel information about spatial and temporal characteristics of CAP states at different frequency bands, which contributes to a better understanding of the frequency aspect of biomarkers for schizophrenia and other disorders.

## Graphical abstract

The resting‐state CAP states have gradually varying spatial and temporal patterns across frequency sub‐bands in the healthy brain. Particularly, CAP states in slow‐4 exhibited stronger coactivation in several subcortical regions than slow‐5. As slow‐5 showed larger inter‐subject differences, slow‐4 achieved a better schizophrenia prediction accuracy.

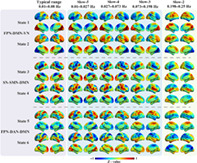

## 1 INTRODUCTION

The resting‐state functional connectivity (RSFC) is temporally varied, which supports that the human brain is a dynamic system (Chang & Glover, 2010). The dynamic functional connectivity (dFC) is generally evaluated by the sliding window approach (Hutchison et al., 2013; Preti et al., 2017). Recurring connectivity configurations can be grouped as FC‐states (Allen et al., 2014) or so‐called brain states, which are related to cognitive and physiological states such as vigilance (C. H. Wang et al., 2016), self‐generated thought (Marusak et al., 2017), eyes open and closed resting state (Weng et al., 2020), as well as disease‐related alterations (Damaraju et al., 2014; Guo et al., 2019; C. Li et al., 2020). However, the sliding window approach is constrained by the choice of window length (Shakil et al., 2016; Zalesky & Breakspear, 2015). Recent work demonstrated that brain states could also be identified based on recurring coactivation patterns (CAPs) from each single frame (Liu et al., 2013; Liu & Duyn, 2013), and reproducible results were achieved in both healthy control and patient groups by using a robust analytical pipeline (Yang et al., 2021).

Besides the temporal dynamics contained in the resting‐state fMRI blood oxygen level dependent (BOLD) signals, frequency‐dependent information also exists. Accumulating evidence demonstrates that fMRI signals and derived measures such as RSFC and network topology have specific properties in the subdivided bands of the conventional low‐frequency band (Ries et al., 2019; Salvador et al., 2008; Thompson & Fransson, 2015). Such frequency‐dependent neural activity and functional brain organization might be attributed to the multiple timescales of complex brain functions (Breakspear, 2017; Buzsaki & Draguhn, 2004; Cocchi et al., 2016; Honey et al., 2007). Therefore, it is important to understand the frequency‐dependent properties of brain states assessed by CAPs. For the fMRI signals with TR of 2 s, based on previous electrophysiological (Buzsaki & Draguhn, 2004) and fMRI studies (Zuo et al., 2010), the frequency range could be subdivided into four frequency sub‐bands including slow‐5 (0.01–0.027 Hz), slow‐4 (0.027–0.073 Hz), slow‐3 (0.073–0.198 Hz), and slow‐2 (0.198–0.25 Hz). Some studies reported that slow‐5 and slow‐4 were more sensitive to detecting disease‐related ALFF changes (Han et al., 2011; Hou et al., 2014; L. Wang et al., 2016), while the other study found the network hub alterations in major depression varied with the frequency sub‐bands from 0.01 to 0.25 Hz (Ries et al., 2019). Since previous CAP studies focused on the low‐frequency band like 0.01 to 0.08 Hz, it remains unknown whether and how the spatial and temporal characteristics of CAPs change in the frequency‐specific manner.

Schizophrenia (SZ) is a severe brain disorder characterized by large‐scale brain changes, including distributed structural loss and functional dysconnectivity (Brandl et al., 2019; Fornito et al., 2012), as well as abnormal dynamic brain states (Hunt et al., 2017; Rashid et al., 2014; Reinen et al., 2018). The literature about dFC in SZ using the sliding window approach has revealed disease‐related abnormal patterns in the time‐varying connectivity, topological properties, and network states (Calhoun et al., 2014; Du et al., 2018; Kottaram et al., 2018; Reinen et al., 2018; Yu et al., 2015), which is not only attributed to the dysfunction of triple networks (including the DMN, CEN, and SN, that is, default‐mode network, executive network, and salience network), for example, reduced dynamic interactions among the DMN, CEN, and SN (Supekar et al., 2019), but also implicates the whole brain dynamic reconfiguration in the brain states disruptions predicting the diagnostic status or active psychotic symptoms (Kottaram et al., 2018; Reinen et al., 2018). Recently, our prior study used the CAP analysis to demonstrate that the altered CAP state dynamics of SZ patients were associated with the triple networks, as well as other primary and high‐order networks (Yang et al., 2021). On the other hand, while the spatiotemporal dynamics of the neural system are manifested at multiple time scales and have the potential to underlie brain disorders (Breakspear, 2017; Honey et al., 2007), only a few studies investigated the frequency‐dependent brain alterations in SZ from the dynamic perspective. For instance, the dFC estimated at different frequency bands helps distinguish SZ patients from healthy controls (Zou & Yang, 2019), slow‐4 and slow‐5 are also linked with distinct dFC strength alterations in SZ (Y. L. Luo et al., 2020). Therefore, an important gap that needs to be filled is whether brain states assessed by CAPs have frequency‐dependent profiles that can better predict the diagnostic status in patients with SZ.

The purpose of this study is to uncover frequency‐specific CAPs, associated abnormalities in SZ, and potential application in patient prediction. So, resting‐state fMRI and four frequency sub‐bands (from slow‐5 to slow‐2 covering 0.01–0.25 Hz) together with the typical low‐frequency range (0.01–0.08 Hz), were analyzed for the spatial and temporal dynamic profiles of brain states in healthy individuals, using a reproducible CAP pipeline (Yang et al., 2021). Particularly, to evaluate the frequency‐specific properties within the typical low‐frequency range, specific CAP spatial profiles and temporal dynamics were further statistically compared between slow‐4 and slow‐5. Next, the frequency‐dependent CAP analysis was conducted in patients with SZ to reveal altered dynamic brain states associated with different frequency bands. Finally, dynamic profiles of CAPs in the sub‐bands and the typical range were implemented to predict the diagnostic status of SZ.

## 2 MATERIALS AND METHODS

### 2.1 Participants

Eighty‐one patients with SZ and one‐hundred healthy controls (HC) were recruited from Wuxi Mental Health Center, Nanjing Medical University. Subjects were excluded if they had any current or past neurological illness, substance abuse or head injury resulting in loss of consciousness, or any MRI contraindications. All the patients met the DSM‐IV‐TR diagnostic criteria (First et al., 2002). The assessments of symptoms were performed on the same day of MRI scanning by experienced psychiatrists using the Positive and Negative Syndrome Scale (PANSS) (Kay et al., 1987). The healthy controls were recruited from the local community via advertisements and free of the history or current diagnosis of any psychiatric disorder. This research was approved by the Medical Ethics Committee of Wuxi Mental Health Center, Nanjing Medical University (study number: WXMHCIRB2012LLKY001), and was conducted in accordance with the Declaration of Helsinki guidelines. Written informed consent was obtained from all participants. Four subjects with excessive headmotion, one subject with failed spatial normalization, and 10 subjects without demographic information were excluded. Thus, 69 SZ patients (35 males/34 females, 46.06 ± 10.96 years) and 97 healthy controls (56 males/41 females, 40.36 ± 14.77 years) remained for the current study. To evaluate the CAPs of each frequency sub‐bands, all the 97 HC subjects were included, in line with our previous work focusing on CAPs of the typical range (Yang et al., 2021). For the group comparison between SZ and HC as well as classification analysis, 69 HC subjects matched for age and gender were used to avoid potential bias in the sample size. There was no significant difference between matched‐HC and SZ concerning the head motion. The detailed demographic information can be found in Table 1.

**TABLE 1:** The demographic information of subjects in the present study

Wuxi	All–HC (*n* = 97)	Matched–HC^a^ (*n* = 69)	SZ (*n* = 69)	*p* value
Age	40.36 ± 14.77	45.84 ± 11.89	46.06 ± 10.96	.9112^b^
Gender (M\F)	56\41	35\34	35\34	1^c^
Mean FD	0.0897 ± 0.0897	0.0842 ± 0.0526	0.0964 ± 0.1048	.2209^d^
Disease duration	—	—	19.84 ± 10.96	—
PANSS positive	—	—	20.06 ± 4.59	—
PANSS negative	—	—	23.78 ± 3.84	—
PNASS general	—	—	41.67 ± 5.27	—
PNASS total	—	—	85.51 ± 9.50	—

*Note*: Data are expressed as mean ± SD.

Abbreviations: SD, standard deviation; FD, framewise displacement indicating the head motion; PANSS, Positive and Negative Syndrome Scale; HC, healthy controls; SZ, patients with schizophrenia.

^a^ The equivalent number of healthy controls were randomly extracted from the HC group and matched for the gender and age with the SZ group, which were utilized in the following group comparisons.

^b^ Two‐sample *t*‐test.

^c^ Chi‐square cross‐table test.

^d^ Permutation test.

### 2.2 fMRI data acquisition and preprocessing

All participants were scanned on a 3T Magnetom TIM Trio (Siemens Medical System) with a 12‐channel phased‐array head coil at the Department of Medical Imaging, Wuxi People's Hospital, Nanjing Medical University. Foam pads were used to reduce head motion and scanner noise. Subjects were instructed to keep their eyes closed, relax but not fall asleep, and move as little as possible during the scanning. Structural MRI images were acquired using a 3D‐MPRAGE sequence (TR/TE = 2530/3.44 ms, flip angle = 7°, FOV = 256 mm, matrix size = 256 × 256, voxel size = 1 × 1 × 1 mm^3^, slice thickness = 1 mm and slice number = 192). Resting‐state fMRI data were obtained using a single‐shot gradient‐echo echo‐planar‐imaging sequence (TR/TE = 2000/30 ms, flip angle = 90°, FOV = 220 mm, matrix size = 64 × 64, voxel size = 3.4 × 3.4 × 4 mm^3^, slice thickness = 4 mm, slice number = 33), and 240 volumes were collected for each subject.

Preprocessing for a reproducible CAP pipeline was conducted according to our prior study (Yang et al., 2021), and the details were described in the Supporting Information. To evaluate the frequency‐dependent CAPs, bandpass filtering was applied to extract fMRI signals in the typical range (0.01–0.08 Hz) and sub‐bands including slow‐5 (0.01–0.027 Hz), slow‐4 (0.027–0.073 Hz), slow‐3 (0.073–0.198 Hz), and slow‐2 (0.198–0.25 Hz) according to the literature (Zuo et al., 2010). The spatial and temporal characteristics of CAPs were computed for each frequency sub‐band and the typical range based on 400 cortical and 8 subcortical ROIs covering the whole brain (Schaefer et al., 2018; Yang et al., 2021). The 400 cortical regions belong to 7 networks, including the visual network (VN), somatomotor network (SMN), dorsal attention network (DAN), ventral attention network (VAN), limbic network, fronto‐parietal network (FPN), and default mode network (DMN).

### 2.3 CAP analysis

The CAP analysis is a data‐driven method that utilizes the K‐means clustering to identify recurring whole‐brain coactivation states, for example, the seed‐and‐threshold free approach (Liu et al., 2013). The robust analytical pipeline was performed to detect the CAP states in different frequency bands (Yang et al., 2021). In brief, there were 235 volumes for each subject's preprocessed fMRI data, and each volume was characterized by the activation level of 408 ROIs. The time series of each ROI was first normalized using z‐score independently, and the absolute value of Z indicated the activation deviation from its baseline. Then, K‐means clustering was performed based on all volumes from the 97 HC subjects, to group volumes sharing similar coactivation profiles into the same CAP state. Our prior study tested the cluster number K from 2 to 21 and identified six robust CAP states in the typical range (Yang et al., 2021). In the present study, we focused on six robust CAPs to compare brain states between sub‐bands and the typical range. Nevertheless, validation analysis also supported that six clusters were suitable for the four frequency sub‐bands based on their silhouette score curves (Figure S1).

For the spatial map of each CAP, volumes belonging to the same state were averaged and divided by their standard deviation to generate a Z‐map (Liu & Duyn, 2013). Pearson correlation was used to measure the spatial similarity between volumes and CAP states. The alignment between CAPs from different frequency bands was conducted by using the Hungarian algorithm (Gutierrez‐Barragan et al., 2019; Kuhn, 1955; Tarun et al., 2020). After determining the CAPs of each frequency band for the HC group, volumes of SZ subjects were assigned to the obtained CAP states based on the highest spatial similarity.

The state temporal dynamic properties of each CAP were also evaluated at the individual level and volume level. For example, if there are total *N* volumes, and *N*_A_ volumes were assigned to State A, then *Fraction of time* which represents the proportion of time occupied by one state, was calculated by *N*_A_/*N*. *Persistence* measures the average time (# of volumes) a state would persist before it transfers to another state, and *counts* records the frequency of one state that occurs across the whole scan. In addition to these state dominances that capture the inner‐state dynamics, the *transition probability* between states was also measured and presented in the Supporting Information. If *N*_A*→*B_ of *N*_A_ volumes make the transition from State A to State B, then the transition probability from state A to state B is calculated as *N*_A*→*B_/*N*_A_. The within‐state transition is also called *resilience*, and it can be extracted from the diagonal of the transition probability matrix (Yang et al., 2021).

### 2.4 Statistical analysis

For the demographic data, two‐sample *t*‐test was used to compare age between SZ and HC, and chi‐square cross‐table test was used to test the group difference in gender. The individual head motion was measured by the mean framewise displacement (FD; Power et al., 2012), and 5000 permutations were used to test its group difference, given the non‐Gaussian distribution.

For the multiband CAPs in the HC group, spatial similarity of each brain state was assessed by Pearson correlation between frequency bands. The repeated‐measures ANOVA was performed to evaluate the effect of frequency bands on temporal dynamics of identified brain states, with the False‐discovery rate (FDR) for multiple comparison correction. As slow‐5 (0.01–0.027 Hz) and slow‐4 (0.027–0.073 Hz) largely constitute the typical low‐frequency range (0.01–0.08 Hz), paired *t*‐test was used to test the ROI‐level differences for each CAP state between slow‐5 and slow‐4 with Bonferroni correction (*p* <.05/408), and to test temporal dynamic differences for each CAP state between slow‐5 and slow‐4 with FDR correction.

The frequency‐specific CAP abnormalities in the SZ group were examined by the group‐by‐frequency interaction using a mixed‐effect ANOVA (SZ vs. HC and slow‐5 vs. slow‐4), with age, gender and mean FD as covariates. For post hoc comparisons, two‐sample *t*‐test (with age, gender, and mean FD controlled) was performed to determine the group differences, and paired *t*‐test was used to detect the frequency effect. FDR correction was performed to account for the multiple comparisons in the ANOVA and post hoc analyses.

### 2.5 Classification analysis

Furthermore, to test if CAP profiles in the frequency sub‐bands can better predict the diagnostic status in patients with SZ, four classification models were built by using spatial features from the typical range, slow‐5, slow‐4, and a combination of slow‐5 and slow‐4. A commonly used machine learning package, LIBSVM (Chang & Lin, 2011) was used to train the SVM classifier with a linear kernel and C = 1. Each subject has 2448 (408 * 6) spatial features, which represents the activation level of the 408 ROIs of the six CAPs. Leave‐one‐pair‐out (LOPO) cross‐validation was used, and accuracy (ACC), sensitivity (SE), specificity (SP), and area under the curve (AUC) were calculated to evaluate the classification performance. For each LOPO iteration, *F*‐score was used for feature selection (Chen & Lin, 2006). Similar to our previous study (H. Yang et al., 2020), the feature number was tested from 20 to 1500 with an incremental step length of 20, and the *F*‐score of all features was first calculated and ranked within the training set, where a larger *F*‐score indicates larger group differences. Then, the smallest step that achieved the highest accuracy was chosen, and the corresponding classification results were reported.

We first used all 69 SZ patients and 69 age/gender‐matched HC subjects (all‐patients model). As the selected pairs were random, the LOPO was repeated 100 times and the classification results were averaged. We also compared the results by using leave‐one‐out (LOO), another popular cross‐validation method. One issue is that the 69 HC subjects were also involved in the CAP definition, which might bias the classification model. Therefore, a hold‐out sample approach was performed to avoid potential bias. Particularly, for the 97 HC subjects, 47 HC subjects were first randomly selected for CAP definition, the remaining 50 HC subjects and 50 age and gender‐matched SZ patients were then used for classification (hold‐out sample model). This random subject assignment process was repeated 100 times, and for each hold‐out repetition, the LOPO was repeated 10 times to get the averaged classification results. The most relevant features contributing to the classification model were also identified. Specifically, there were 50 LOPO iterations for each hold‐out repetition, as each pair of subjects would serve as the testing set in turn and the other 49 pairs were involved in the training set. The LOPO was repeated 10 times, hence, there were a total of 500 iterations for each hold‐out repetition. The *F*‐score was used to select features within each iteration, and features that were chosen in 80% of the 500 iterations were flagged. Then, features that were flagged among 80% of the 100 hold‐out repetitions were further extracted as the consensus features. Finally, an independent dataset, the Center for Biomedical Research Excellence (COBRE), was used to test the generalizability of our classification method. We mainly reported the results based on the hold‐out sample approach in the main text. More details and results are described in the Supporting Information.

## 3 RESULTS

### 3.1 Spatial and temporal properties of CAPs at different frequency bands

**FIGURE 1:**
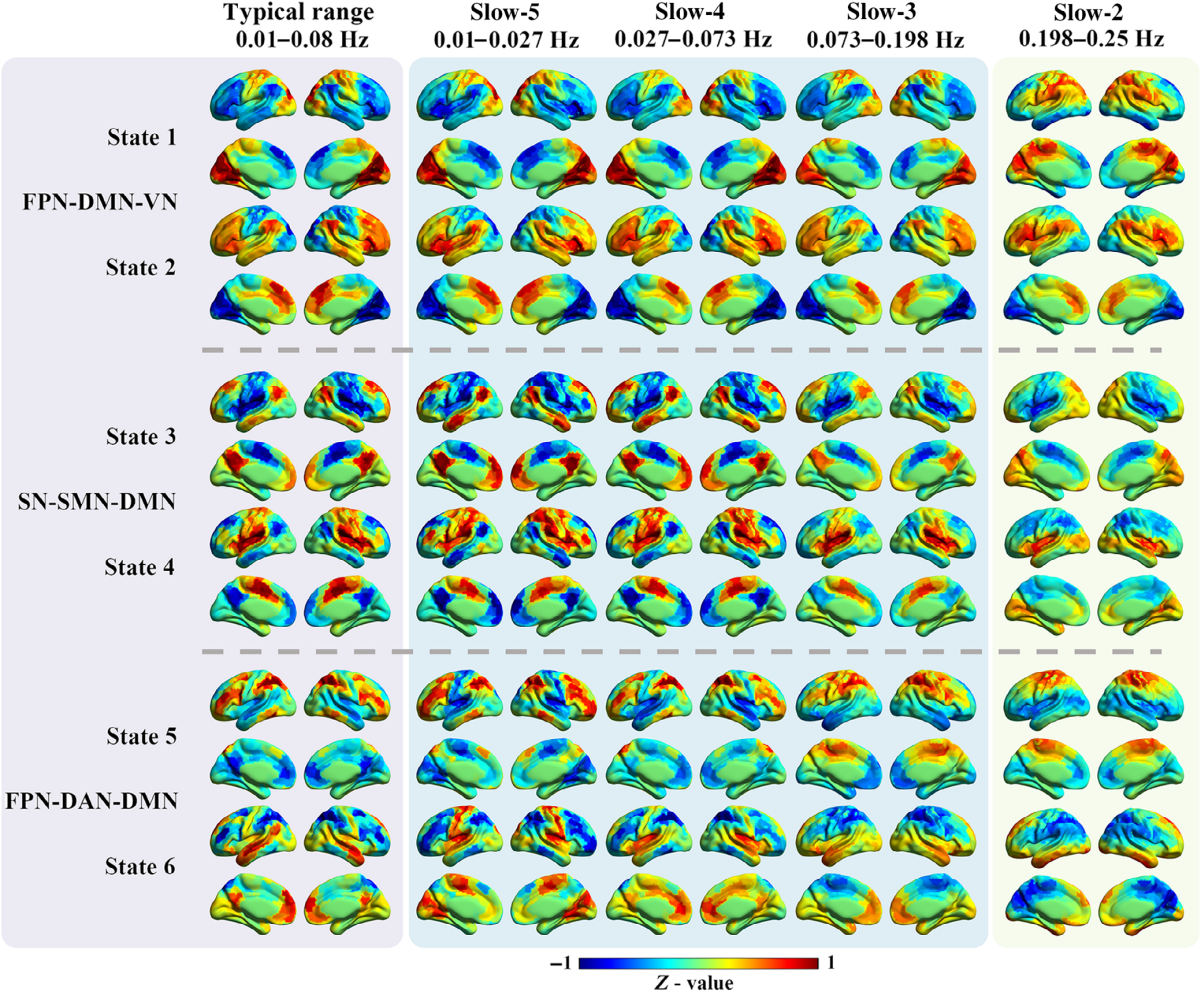
The spatial patterns for the six CAP states in different frequency bands. The first column shows the six CAP states in the typical range, and the six states were grouped into three pairs with opposite coactivation profiles. The following four columns show the six CAP states from slow‐5 to slow‐2. For each ROI, the *Z*‐value means the degree of activation deviation from its baseline. The warm color indicates a relatively stronger BOLD response than its baseline amplitude, and vice versa for the cold color. DAN, dorsal attention network; DMN, default mode network; FPN, fronto‐parietal network; SN, salience network; SMN, somatomotor network; VN, visual network

**FIGURE 2:**
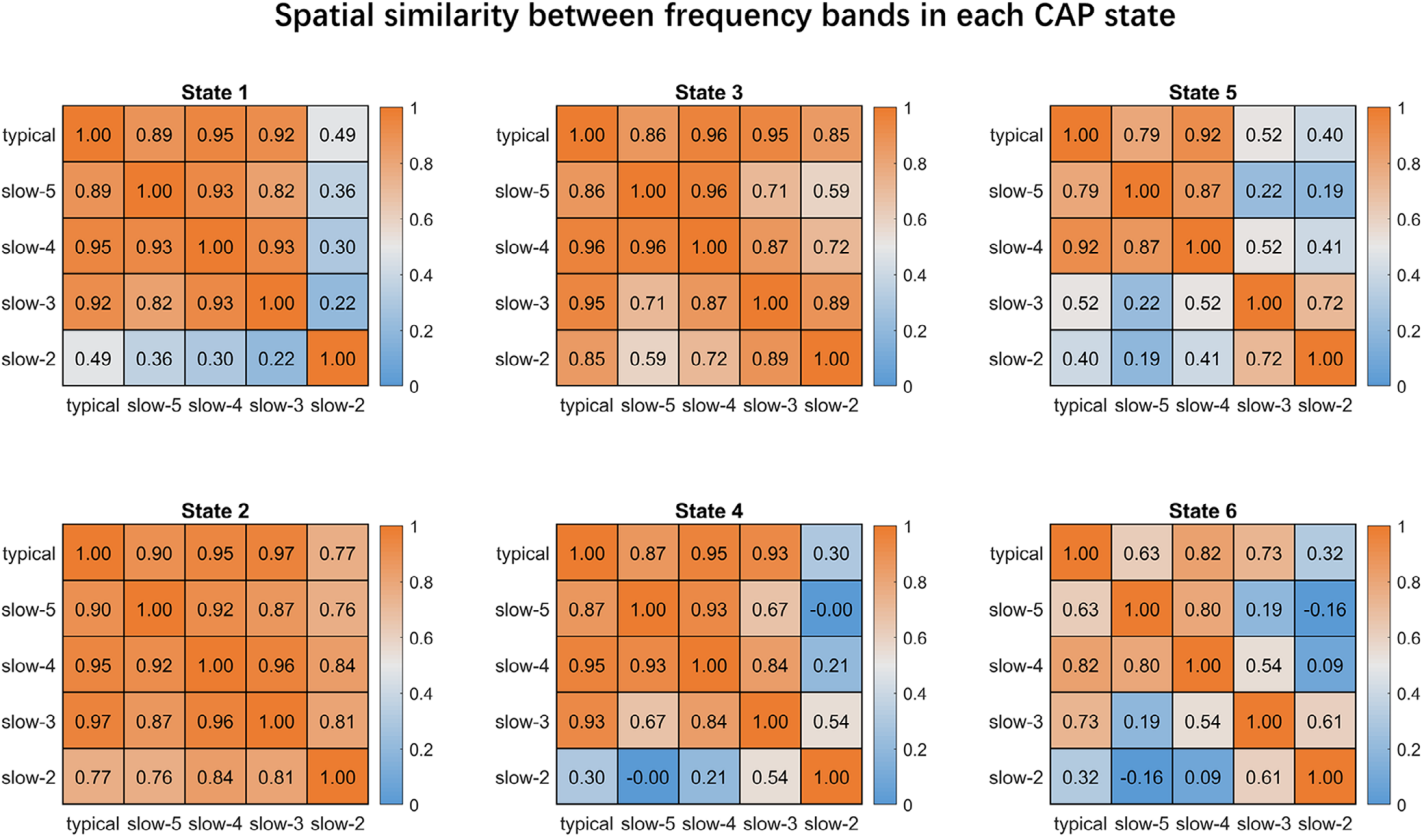
The coactivation pattern (CAP) spatial similarity between the typical range and four sub‐bands. The six CAP states of the sub‐bands were first matched with those of the typical range, and then assessed for between‐band similarity of each CAP by Pearson correlation. The colorbar shows the *R*‐value

The CAP analysis was performed using all 97 HC subjects in the typical range and four frequency sub‐bands (slow‐5 to slow‐2) separately. In total three pairs of CAPs, spatially matched across the typical range and four sub‐bands, were identified and shown in Figure 1. Typical intrinsic high‐order (e.g., FPN and DMN) and primary networks (e.g., VN and SMN) can be observed in the typical range, slow‐5, slow‐4, and slow‐3, but the high‐order networks were absent in slow‐2. For instance, the DMN and FPN cannot be found in any state in slow‐2. The overall spatial similarity of CAP states was high between the typical range, slow‐5 and slow‐4, and became lower in slow‐3 and slow‐2 (Figure 2). Particularly for State 5 and 6, only the typical range, slow‐5 and slow‐4 showed high spatial similarities between each other. The highest spatial similarity with the typical range was found in slow‐4 for all six CAP states except slow‐3 for State 2. Besides, slow‐5 also displayed high spatial similarity with the typical range for all CAP states, and slow‐2 was similar to the typical range only for State 2 and State 3 (higher than 0.5).

The time series of each ROI was normalized by using Z‐score, and the mean of the time series represented its baseline activation level (*Z* = 0). As the existence of opposite CAP pairs, the absolute *Z* value of each ROI indicated the amplitude of deviation from the baseline, and was defined as activation deviation in this work. A larger activation deviation means a stronger positive activation or stronger negative deactivation. For example, in the typical range, compared with other brain areas, regions within the visual network exhibited stronger positive activation in State 1, and stronger negative deactivation in State 2 (Figure 1). Hence, State 1 and State 2 showed larger activation deviations in the visual network, and the visual network was the dominant network for State 1 and State 2.

In our previous study, the six CAP states were grouped into three pairs in the typical range, and the paired CAP states were characterized by opposite coactivation profiles (Yang et al., 2021). In the present study, CAP states were identified and matched for both the typical range and sub‐bands, which were three paired states. For instance, State 3 and State 4 belong to an opposite CAP pair. The DMN was activated, and the SMN and SN were deactivated in State 3, while the DMN was deactivated, and the SMN and SN were activated in State 4. Despite becoming weaker from the typical range and slow‐5 to slow‐2, State 1 and State 2 were mainly dominated by the VN, FPN, and DMN, State 3 and State 4 were mainly dominated by the SN, SMN, and DMN, while State 5 and State 6 were mainly dominated by the FPN, DAN, and DMN, in line with our prior work (Figure 1). Furthermore, the between‐state spatial similarity matrix was measured for each frequency sub‐band, respectively in this study. On the one hand, strong opposite patterns (Pearson correlation < −.95) can be observed for CAP pairs in slow‐5, slow‐4, and slow‐3, similar to those of the typical range; on the other hand, slow‐2 displayed distinct and weaker between‐state spatial similarity, which was dissimilar with those of the typical range (Figure S2). Additionally, paired CAP states with strong opposite patterns showed the lowest transition probability between each other in slow‐5 and slow‐4, consistent with results of the typical range reported in the previous study. But the relationships between CAP pairs, spatial similarity and transition probability differed in slow‐3 and slow‐2 (Figure S4).

**FIGURE 3:**
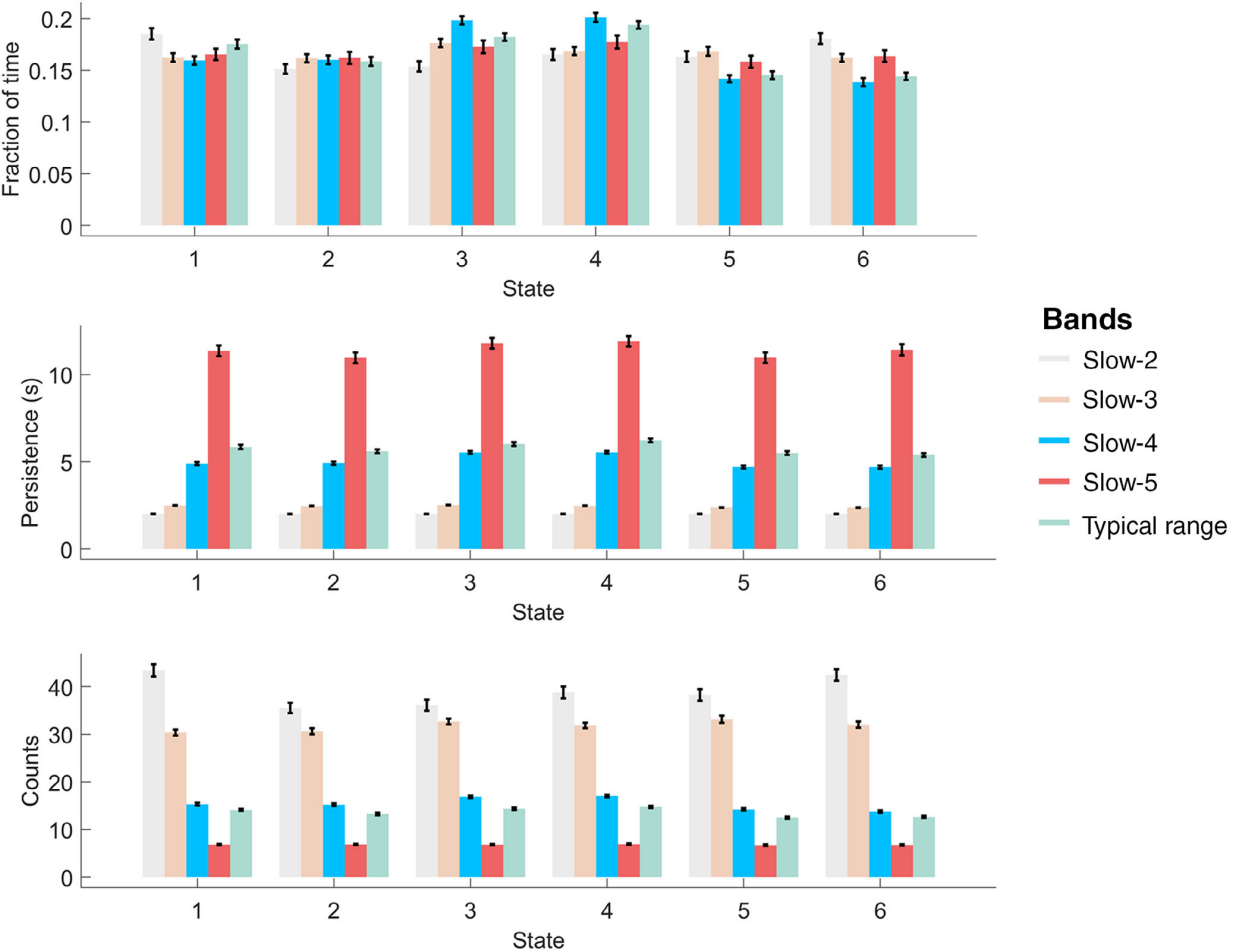
The state temporal dominances (fraction of time, persistence, and counts) in the typical range and four frequency sub‐bands. The error bar shows the standard error. One‐way repeated‐measures ANOVA was performed to evaluate the frequency‐specific dynamics between the four sub‐bands. Except for fraction of time in State 2, all dynamic metrics showed significant frequency effects (*p* <.0001, FDR adjusted) in the six states

The repeated measures ANOVA results supported the significant frequency effects on the temporal dynamics of CAP states (Figure 3). All CAP states except State 2 showed significant frequency effects in fraction of time, and all the six CAP states showed significant frequency effects in persistence and counts (*p* <.0001, FDR adjusted). The mean fraction of time was comparable across the six CAP states for all frequency bands, around 15%–20%. For the persistence and count, as the frequency decreased from slow‐2 to slow‐5, each state had longer and longer dwell time before it transferred to another state, and its count decreased correspondingly. For example, each state persisted for only 2 s in slow‐2 and slow‐3, but about 5 s for slow‐4 and 12 s for slow‐5. The persistence and count of the typical range were between those of slow‐4 and slow‐5, and similar to slow‐4, which might suggest the crucial role of sub‐band slow‐4 in the intrinsic neural information.

### 3.2 Specific spatial and temporal characteristics of CAPs in slow‐4 and slow‐5

**FIGURE 4:**
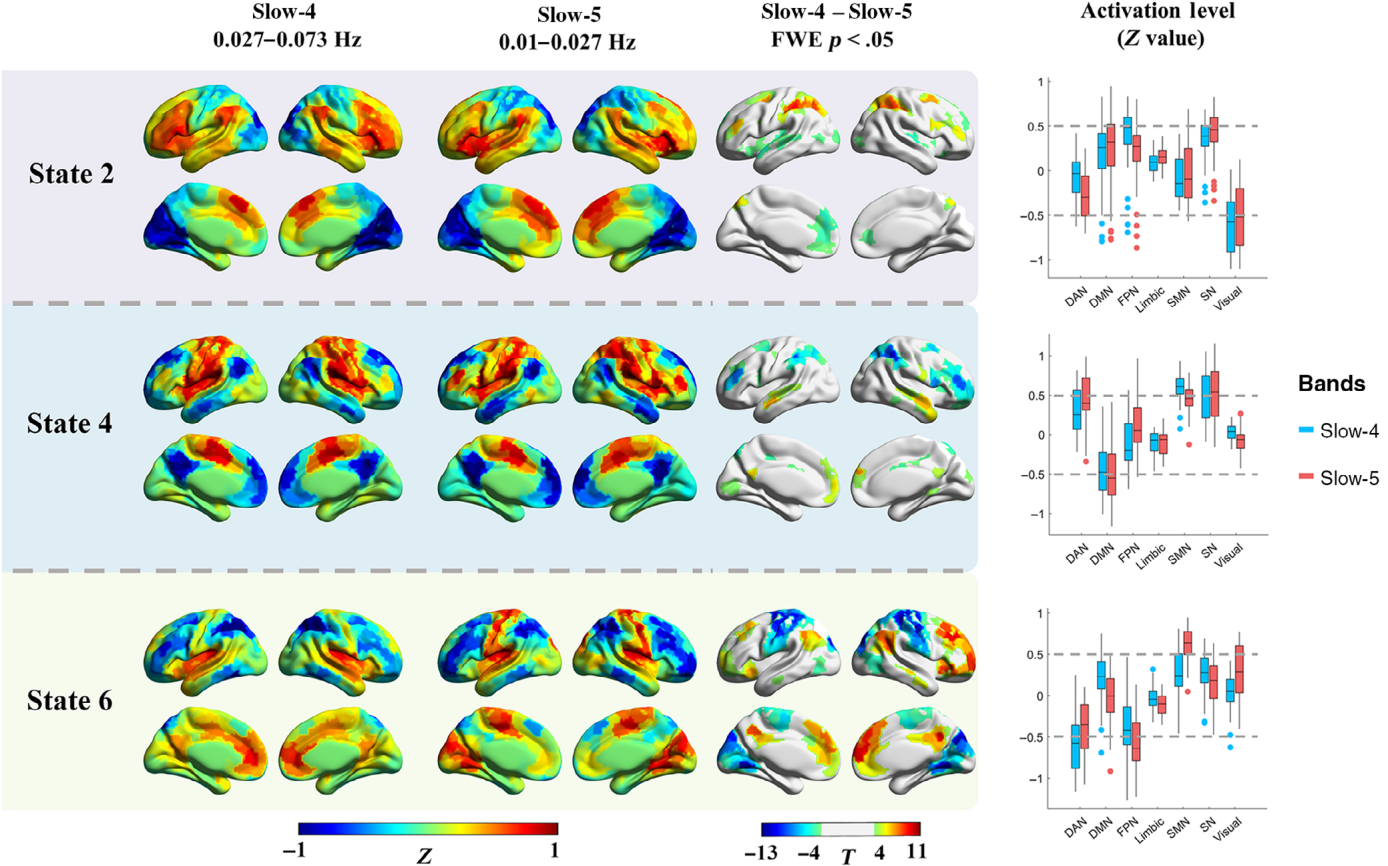
The frequency‐specific effects between slow‐4 and slow‐5 within the HC group. The results of three states were presented, as the six CAP states were grouped into three pairs, and similar results were found within the pair. The first two columns show the cortical coactivations, and the color of each ROI indicates the activation deviation from its baseline level (*Z*‐value). Paired *t*‐test was performed for each state separately, and Bonferroni correction was used at the ROI level. The colorbar shows the *T*‐value, and regions with *p* <.05 (FWE corrected) were presented in the third column. The last column shows the activation level of the seven networks in slow‐4 and slow‐5, and each point represents an ROI's group averaged activation level from all 97 HC subjects

**FIGURE 5:**
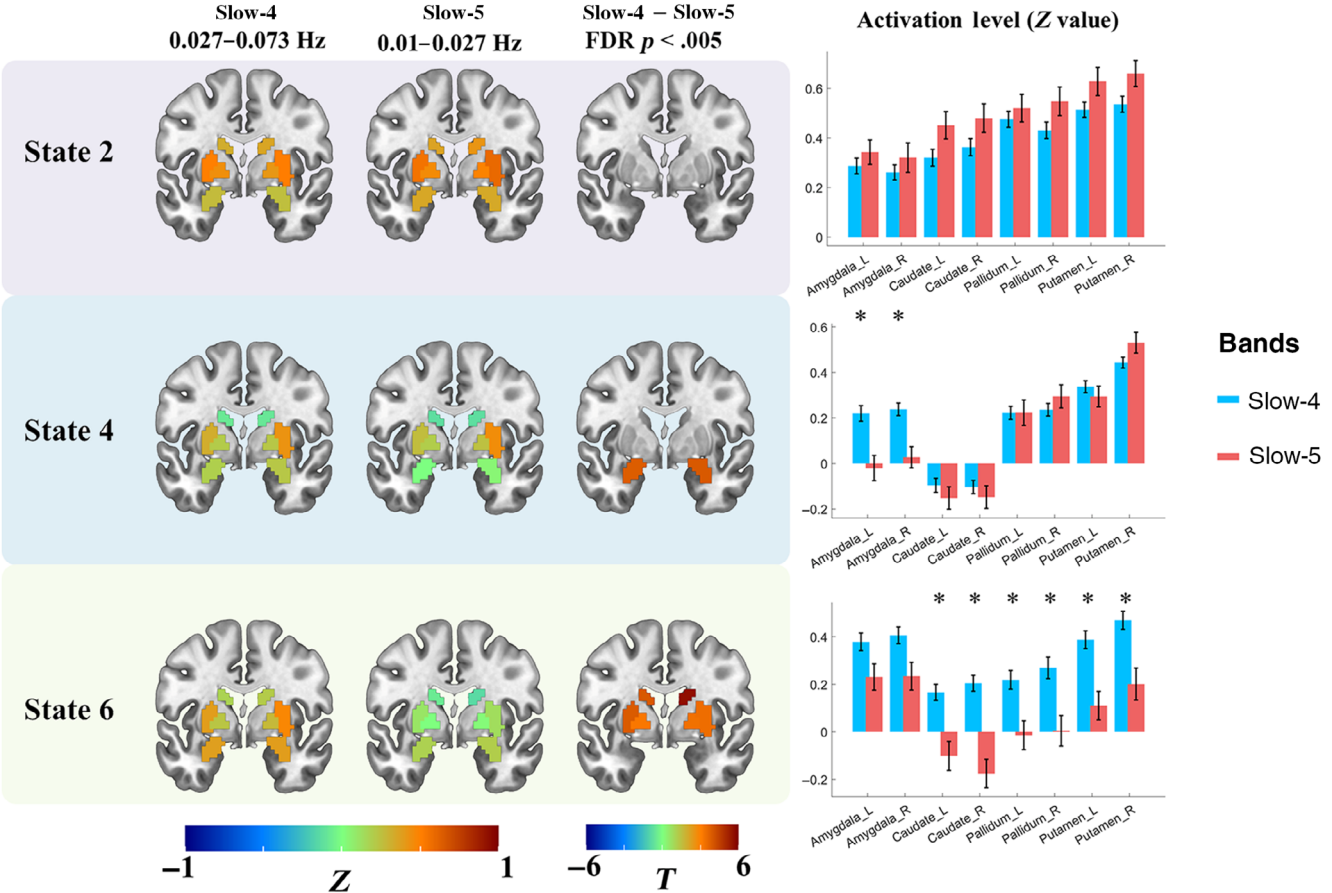
The subcortical activation differences between slow‐4 and slow‐5 within the HC group. The first two columns show the coactivation level of the eight subcortical regions, and the color of each ROI indicates the activation deviation from its baseline level (*Z*‐value). Paired *t*‐test was performed for six states separately, and FDR correction was used at the ROI level. Regions with *p* <.005 (FDR adjusted) were presented in the third row, and the colorbar shows the *T*‐value. The last column shows the activation level of the eight subcortical regions in slow‐4 and slow‐5, from all 97 HC subjects. “*” indicated FDR adjusted *p* <.005

While slow‐4 and slow‐5 constitute most power of the low‐frequency oscillations, they represent the intrinsic neural activities that have been intensively studied in resting‐state fMRI studies, and are less likely to be as affected by non‐neural noise (e.g., heartbeat and respiration) as higher frequency sub‐bands (e.g., slow‐2). Therefore, in the current analysis, we mainly focused on the frequency‐specific effects on CAPs in slow‐4 and slow‐5. While high correspondence of CAPs between slow‐4 and slow‐5 was found, as shown in the main diagonal of the spatial similarity matrix (Figure S5), paired *t*‐test results of CAPs between slow‐4 and slow‐5 within the HC group showed frequency‐specific spatial and temporal characteristics for sub‐bands within the typical low‐frequency range (0.01–0.08 Hz). Because paired CAPs had opposite CAPs (State 1 and 2, State 3 and 4, State 5 and 6), here we presented results of State 2, 4, and 6 for simplicity (Figures 4 and 5), results of State 1, 3, and 5 can be found in the Figures S6 and S7.

As shown in the first two columns in Figure 4, the activation level was represented by the Z value. Then the group averaged activation level of the 408 ROIs in slow‐4 and slow‐5 were categorized into seven networks by using boxplots and represented in the last column. State 2 was dominated by large activation deviation in the VN, as well as SN and FPN. State 2 in slow‐4 showed higher activation deviation in the bilateral middle frontal gyrus (FPN), as well as lower activation deviation in the anterior DMN, bilateral insula (SN), and dorsal attention network (DAN), than slow‐5. While State 4 was dominated by the SN, SMN, and DMN, slow‐4 showed lower activation deviation in the DMN and FPN than slow‐5. For State 6, large DAN and FPN activation deviation was found in both slow‐4 and slow‐5, while slow‐5 was also dominated by the SMN. State 6 in slow‐5 showed significantly lower activation deviation in the SMN and VN, and higher activation deviation in the DMN and FPN. Meanwhile, subcortical regions also showed different CAP states between slow‐4 and slow‐5. Particularly, slow‐4 exhibited an overall stronger subcortical activation deviation than slow‐5. Though no significant result was found in State 2, slow‐4 showed stronger activations for the bilateral amygdala in State 4, and stronger activations for the bilateral basal ganglia (caudate nucleus, putamen, and globus pallidus) in State 6 (Figure 5). On the other hand, slow‐4 showed weaker deactivations for bilateral globus pallidus and left putamen in State 1, stronger deactivation for bilateral striatum and amygdala in State 3, as well as stronger deactivation for right striatum in State 5 (Figure S7).

Moreover, significant differences between slow‐4 and slow‐5 were also found for the CAP temporal dynamics within the HC group (Figure S8a). Compared with slow‐5, all six states showed significantly shorter persistence and more counts in slow‐4. More fraction of time in State 3 and State 4, and less fraction of time in State 5 and State 6 were observed in slow‐4. In addition, the variation of fraction of time between the six states was also evaluated in slow‐4 and slow‐5 separately (Figure S8b). No between‐state difference was found for fraction of time in slow‐5, in which each state occupied about 15%–17% of the time. However, significant between‐state differences were found in slow‐4, for example, State 3 and 4 showed more fraction of time (about 20%) than the other four states.

### 3.3 Frequency‐specific CAP dynamic alterations in SZ

**FIGURE 6:**
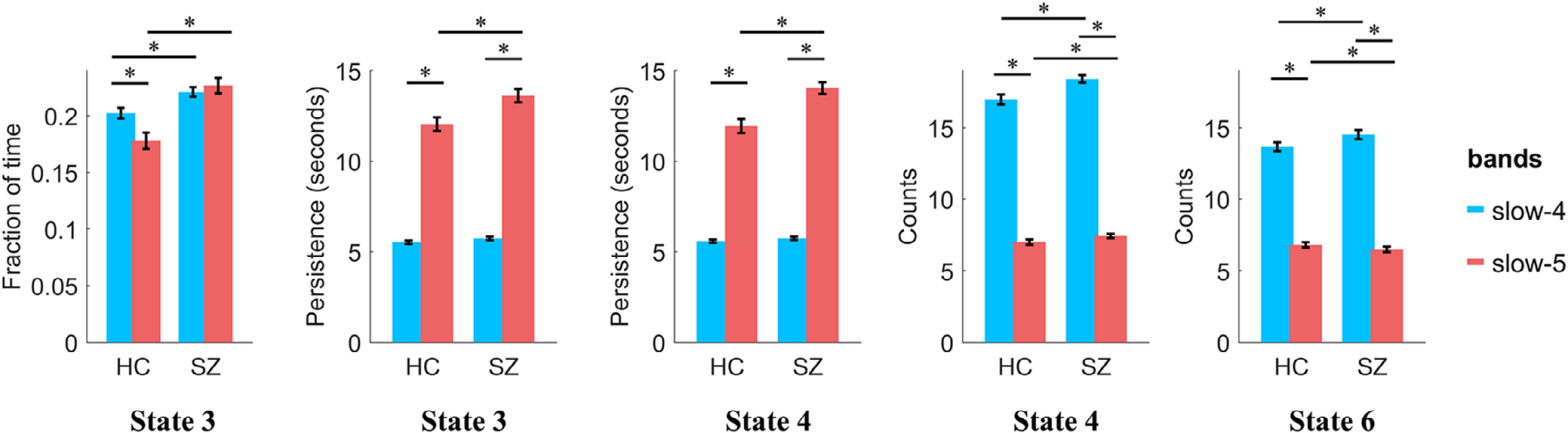
The post hoc results of mixed‐effect ANOVA. Only the results with significant interaction effect were compared. Between‐group differences were compared using two‐sample *t*‐test, and between frequency differences were compared using paired *t*‐test. Age and gender were controlled for between‐group comparisons. FDR correction was performed to correct the multiple comparisons. Error‐bar shows the standard error. “*” indicates *p* <.05 with FDR correction

Since differences of six CAPs between SZ and HC in the typical range have been examined in our prior work (Yang et al., 2021), the current study further examined between‐group differences in slow‐4 and slow‐5, focusing on the frequency‐specific CAP dynamic alterations by mixed‐effect ANOVA and group‐by‐frequency interaction. The detailed statistic results were presented in Table S5. For significant main effects of group, SZ showed decreased fraction of time in State 1 and State 2, and increased fraction of time in State 3 and State 4. SZ also showed deceased persistence in State 2, and increased persistence in State 3 and State 4. Finally, deceased counts in State 1 and State 2, and increased counts in State 3 and State 4 were observed in SZ. The significant main effect of frequency on fraction of time was also found. Fraction of time increased in State 3 and State 4 and decreased in State 5 in slow‐4. For persistence and counts, significant main effects of frequency were found in all six states. As described before, higher‐frequency (slow‐4) CAPs showed shorter persistence and more counts than lower‐frequency (slow‐5). Importantly, significant frequency‐by‐group interaction was found for fraction of time (*p* = .0015, *F* = 10.55) in State 3, persistence in State 3 (*p* = .0089, *F* = 7.05), and State 4 (*p* = 1.51 × 10^−4^, *F* = 15.20), counts in State 4 (*p* = .0408, *F* = 4.27), and State 6 (*p* = .0264, *F* = 5.04). Post hoc results are illustrated in Figure 6, SZ showed increased fraction of time in State 3 in both slow‐4 (*p* = .0080, *T* = 2.82) and slow‐5 (*p* = 3.93 × 10^−6^, *T* = 5.00). Significant group differences in counts in State 4 and State 6 were also found in both slow‐4 and slow‐5. But only in slow‐5, we found significant group differences of persistence, for example, SZ showed increased persistence in both State 3 (*p* = .0039, *T* = 3.08) and State 4 (*p* = 1.46 × 10^−4^, *T* = 4.09).

### 3.4 SZ classification based on CAPs in different frequency bands

**FIGURE 7:**
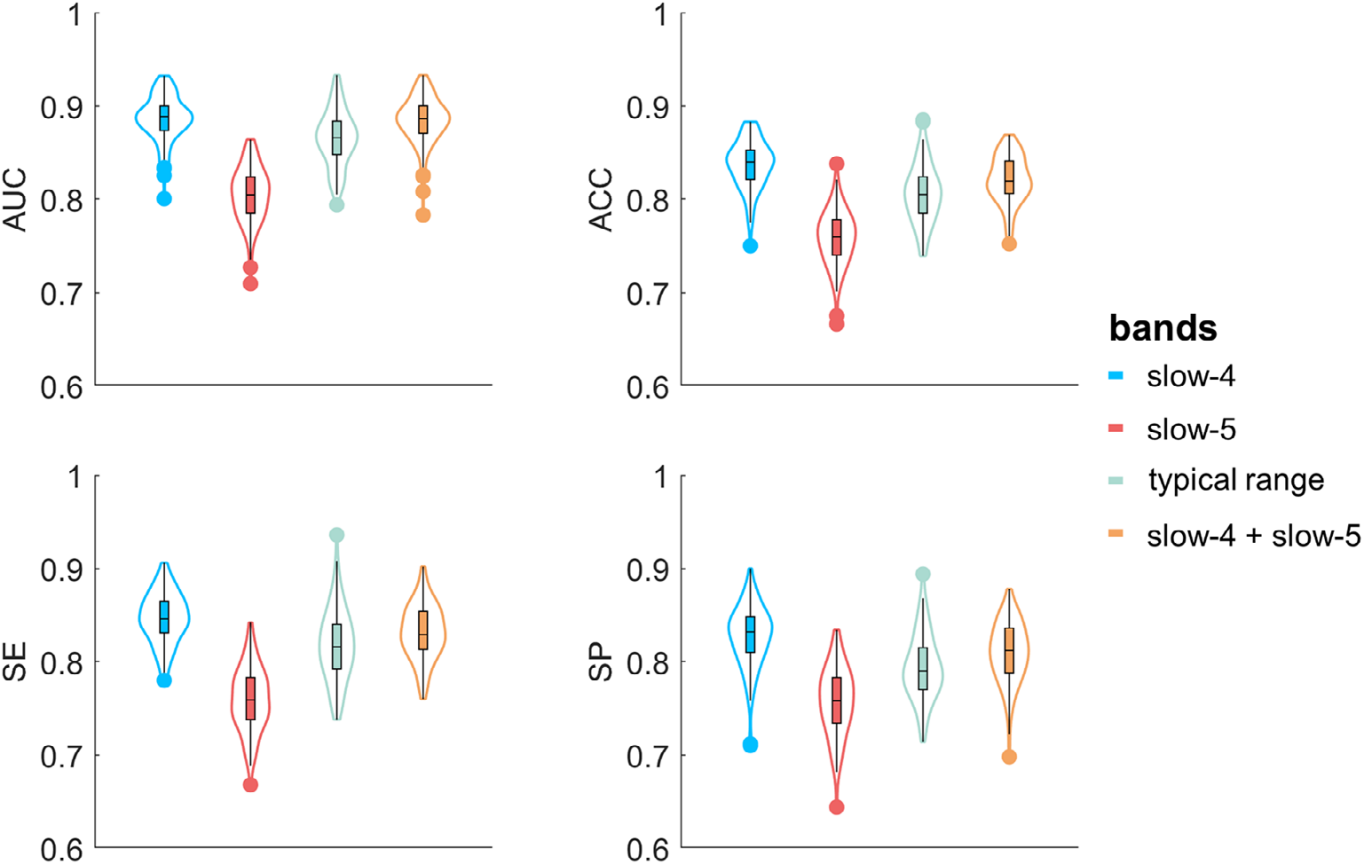
The classification results of the 100 hold‐out repetitions. For the 97 HC subjects, 47 HC subjects were randomly selected to define the CAPs, and the remained 50 HC subjects and age/gender‐matched SZ patients were used for classification. This procedure was repeated 100 times, and each point within the violin plot represents the result of one repetition. ACC, accuracy; AUC, area under curve; SE, sensitivity, SP, specificity

**FIGURE 8:**
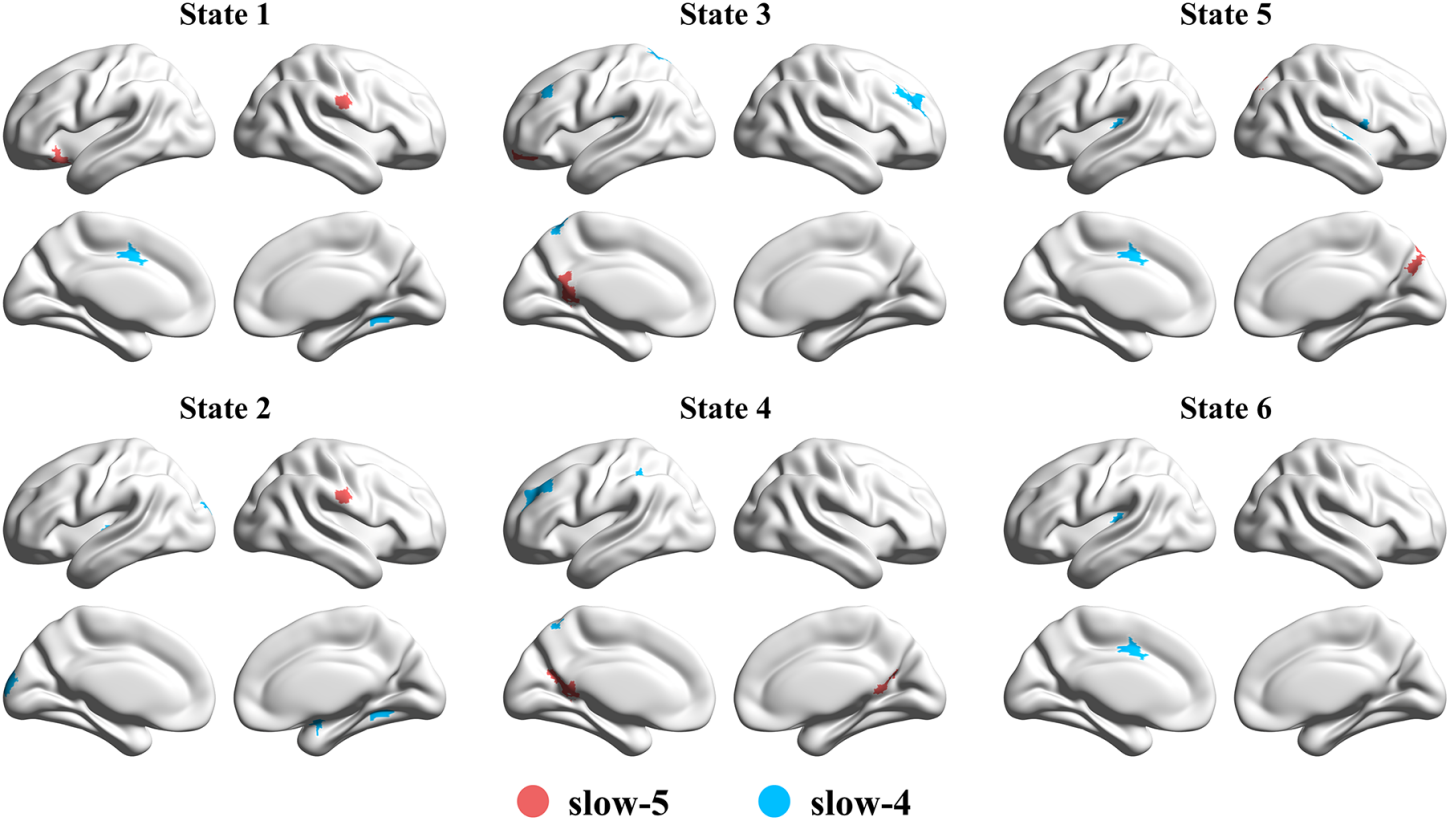
The consensus features of the combined model (slow‐5 + slow‐4). The red color indicates the unique feature from slow‐5, and the blue color indicates the unique feature from slow‐4. No intersection was found between slow‐5 and slow‐4

To address our hypothesis that sub‐band information might help predict the clinical diagnosis of SZ, classification was carried out based on spatial features of CAPs in sub‐bands and was compared with the typical range. Models that used all the 69 SZ patients and 69 matched HC subjects achieved the classification accuracy of ~85%–90% when using either LOPO or LOO cross‐validation (Table S6). Combining slow‐4 and slow‐5 slightly increased the accuracy than using the single sub‐frequency band. Notably, although machine learning (including feature extraction and cross‐validation based on whole samples) is widely utilized in neuroimaging studies and clinical patient diagnosis prediction, our initial models might be susceptible to potential bias as the HC subjects were also involved in the CAP definition. Therefore, a hold‐out sample approach was used for reporting our main results (see Section 2). The classification results based on the 100 hold‐out repetitions are shown in Figure 7, and their average values are presented in Table 2. Typically, the accuracy decreased by about 5%–10%, and slow‐4 itself, rather than combining slow‐4 and slow‐5, achieved the highest accuracy (~84%), while slow‐5 had the lowest accuracy (~75%). In addition, several brain regions associated with multiple CAP states as well as slow‐4 and slow‐5 were identified as consensus features (Figure 8). It can be observed that slow‐5 and slow‐4 provided unique features across the six CAP states. For instance, for State 3 and 4, the consensus features were mainly located at the bilateral middle frontal gyrus and left superior parietal lobe in slow‐4, and the left precuneus in slow‐5, respectively. Finally, the validation analysis was performed in the independent COBRE dataset (Table S7). Compared to the results obtained by the Wuxi dataset, all the four models based on the COBRE dataset exhibited lower classification accuracy, and the highest accuracy was obtained in the typical range (AUC = 0.7430, ACC = 0.6953, SE = 0.7032, SP = 0.6874).

**TABLE 2:** The averaged classification results (50 SZ vs. 50 HC, 100 repetitions)

	Typical range	Slow‐5	Slow‐4	Slow‐5 + slow‐4
AUC	0.8653	0.8050	0.8861	0.8842
ACC	0.8059	0.7583	0.8369	0.8207
SE	0.8176	0.7591	0.8470	0.8315
SP	0.7942	0.7575	0.8268	0.8099

Abbreviations: ACC, accuracy; AUC, area under curve; SE, sensitivity; SP, specificity.

## 4 DISCUSSION

The current study focused on the frequency‐specific CAPs in healthy adults, and frequency‐specific abnormalities in SZ, by combining statistical comparison and classification analysis. Specifically, similar CAP spatial patterns with the typical range were found in slow‐5, 4, and 3, but not in slow‐2, regarding both the high‐order and primary networks. As the frequency increased, CAPs showed subtle spatial alterations, as well as shorter persistence and more occurrence at higher frequency sub‐band. Slow‐4 was associated with stronger coactivation in the basal ganglia as well as amygdala than slow‐5, in addition to divergent CAP changes in the cortical networks. Moreover, SZ patients demonstrated frequency‐specific CAP persistence abnormalities in slow‐5. Meanwhile, higher accuracy for patient classification was achieved by using CAP spatial features of slow‐4. To the best of our knowledge, our findings provide the first evidence for frequency‐specific CAPs in the resting state and their alterations in SZ.

### 4.1 Spatial and temporal properties of CAPs in different frequency bands in the healthy controls

The current study delineated both spatial and temporal properties of six CAP states in each of the sub‐bands from slow‐5 to slow‐2 (Figures 1 and 3), which extended our understanding of CAPs in the typical range (0.01–0.08 Hz) as reported in our prior study (Yang et al., 2021). Using a reproducible CAP analytical pipeline (Yang et al., 2021), our findings demonstrated that CAP states of the healthy adults in sub‐bands were similar to some extent to those in the typical range. Concerning the spatial maps of all CAP states, slow‐4 (0.027–0.073 Hz) showed the highest similarity with the typical range, although in State 2 highest similarity with the typical range was found in slow‐3 (0.073–0.198 Hz; Figure 2). This might be due to the large frequency overlap between slow‐4 and the typical range sharing the neural fluctuations related to intrinsic brain networks. Both slow‐5 (0.01–0.027 Hz) and slow‐4 (0.027–0.073 Hz) displayed highly similar spatial patterns with the typical range, in which CAP states were dominated by intrinsic functional networks including triple‐network of DMN, FPN, and SN (Menon, 2011; Yang et al., 2021). Additionally, opposite spatial patterns for paired CAP states were pronounced in sub‐bands, which was consistent with previous literature (Huang et al., 2020; J. Zhang et al., 2020), and suggested that the antagonistic relationships between these intrinsic networks widely exist in different frequency bands.

While slow‐3 seemed to be the transient sub‐band, slow‐2 (0.198–0.25 Hz) and slow‐3(0.073–0.198 Hz) had a lower degree of similarity for CAP states than other sub‐bands as well as the typical range, together with shorter persistence and larger counts for each CAP state across all bands (Figures 2 and 3). This might be because slow‐2 and slow‐3 are the high‐frequency sub‐band of current fMRI signals with TR of 2 s, contrary to the typical range for identifying intrinsic and dynamic brain networks (Allen et al., 2014; Biswal et al., 1995). Particularly, the CAP states in slow‐2 (0.198–0.25 Hz) were unlike those of the typical range, meaning brain regions of specific brain networks were not coactivated but mixed with those of other networks or even dominated by white matter signals. A previous resting‐state fMRI study found that slow‐2 oscillated within white matter rather than gray matter (Zuo et al., 2010). The seed‐based FC maps in slow‐2 showed significantly reduced spatial extent compared with lower frequency sub‐bands (Gohel & Biswal, 2015). Since slow‐2 is more susceptible to high‐frequency neurophysiological signals as well as non‐neuronal noise (Chen & Glover, 2015; Cordes et al., 2001; Keilholz et al., 2017), functional networks and dynamic brain states are mainly associated with frequency sub‐bands of slow‐5 to slow‐3, and have attenuated association with slow‐2.

As for the temporal dynamics of CAP states, persistence and counts changed monotonically with the increased frequency band. Particularly, persistence decreased, and counts increased for all the six states from slow‐5 to slow‐2, suggesting that the higher frequency leads to faster state transition or unstable state maintenance. First, the higher frequency could cause more frequent BOLD fluctuations, hence the volume‐to‐volume state maintenance would decrease, and the between‐state transition would increase. As the fraction of time was similar across different frequency bands, shorter persistence led to more counts. Besides, the higher frequency BOLD signal involved more noise (Chen & Glover, 2015; Cordes et al., 2001), which might contaminate and mislabel the coactivation profile and result in more between‐state transitions, and shorten the persistence.

In addition, to test whether the frequency‐dependent persistence was only due to the varied filtering procedures, we normalized the persistence based on its center frequency (Tables S8 and S9). As shown in Figure S13, the normalized persistence across frequency bands was still different, suggesting the effect of filtering was nonlinear. Although slow‐4 and slow‐5 showed similar normalized persistence, the differences were still significant. More details can be found in the Supporting Information. How filtering procedure and other factors affect the temporal dynamics remains a topic for further investigation.

### 4.2 Subtle but significant frequency‐dependent effects on spatial and temporal configurations of CAPs in slow‐4 and slow‐5

Focusing on the low‐frequency bands, frequency‐specific coactivation profiles were found by comparing CAP states between slow‐5 and slow‐4. The current study revealed subtle but significant CAP differences in distributed cortical networks and basal ganglia as well as the amygdala (Figures 4, 5, S6, and S7). For cortical networks, higher‐order networks (DMN, FPN, SN, and DAN) and primary networks including the VN and SMN showed significantly different coactivation or de‐coactivation in their dominant CAP states, which extended previous evidence about greater ALFF/fALFF of DMN in slow‐5 (Han et al., 2011; L. Wang et al., 2016). On the other hand, subcortical regions demonstrated overall stronger activation deviations in slow‐4 than slow‐5 for most CAP states. For instance, State 6 in slow‐4 showed stronger activation at bilateral basal ganglia, which was consistent with previous findings that stronger basal ganglia ALFF/fALFF in slow‐4 (Zuo et al., 2010). This might be owing to the influence of different ranges on FC and dynamics. Previous literature has revealed that large‐scale functional networks (e.g., DMN and FPN) integrate remote brain regions with long‐range interactions, while subcortical regions are spatially compact and dominated by local neural activities (Salvador et al., 2005). Seemly, short‐range functional interaction relies on the higher‐frequency band of slow‐4 (Buzsaki & Draguhn, 2004). It is worth noting that, the current study provided new evidence about the subcortical CAP profiles in slow‐4, since previous studies examined only the static measures of slow‐4 (Zuo et al., 2010) or extremely high‐frequency band (0.3032–0.4545 Hz) (Salvador et al., 2005). Together, current findings elucidated carefully CAP states in sub‐bands within the typical low‐frequency range, which suggest frequency‐specific information in the dynamic brain organization.

As for the temporal domain, slow‐4 showed significantly shorter persistence and more counts across all the six CAPs, while slow‐5 had long persistence (Figure 3) and high within‐state transition probability (Figure S3) as well as the between‐state balanced fraction of time (Figure S8b). Faster signal change and functional interaction are postulated to link with slow‐4 (Buzsaki & Draguhn, 2004), which could help explain the phenomena of higher frequency band shorter persistence for CAP states. But it remains unclear why some CAP states, for example, the pair of State 3 and 4, which were dominated by the DMN, SN, and SMN, occurred more in terms of the increased fraction of time, in slow‐4 than slow‐5. Probably, as the frequency increases from slow‐5 to slow‐4, the DMN is more frequently recruited for ongoing functional processes. If so, slow‐4 might provide more information about the dynamics of DMN, supporting that DMN has frequency‐specific properties (Park et al., 2019). Nevertheless, future study is needed to investigate the electrophysiological foundation of DMN (Das et al., 2020; Samogin et al., 2019).

### 4.3 Frequency‐specific CAP abnormalities in SZ

The current study revealed significant frequency‐by‐group interaction on CAP state temporal profiles by using mixed‐effect ANOVA (Table S5). Post hoc results suggest that SZ link with abnormal CAP states commonly in slow‐4 and slow‐5 (Figure 6). Notably, our findings based on classification demonstrated that, slow‐4 and slow‐5 provided distinct CAP features for the clinical prediction (Figure 8). Together, CAP alterations in SZ patients based on frequency sub‐bands enhance our understanding of abnormal dynamics of brain networks in SZ.

First, altered state temporal dynamics of CAPs such as fraction of time, persistence and count, were identified in slow‐4 and slow‐5 (Tables S3–S5), which are similar to changes in the typical range reported in our prior study (Yang et al., 2021). Accumulating work also pointed out that SZ patients are not only characterized by frequency‐specific changes (Gohel et al., 2018; Yu et al., 2013; Y. Zhang et al., 2020) or temporal dynamic changes (Du et al., 2017; Kottaram et al., 2018), but also frequency‐specific dynamic alterations (Y. L. Luo et al., 2020; Zou & Yang, 2019). Second, in both sub‐bands, fraction of time decreased in the FPN‐DMN state (State 1 and 2) and increased in the SN‐DMN state (State 3 and 4) in both slow‐4 and slow‐5, supporting the triple‐network model in SZ (Manoliu et al., 2014; Menon, 2011; Supekar et al., 2019). Third, frequency‐specific properties were represented by significant group‐by‐frequency interactions in the SN‐DMN state (State 3 and 4) including the abnormal fraction of time, persistence and counts. Particularly, the persistence of the SN‐DMN state significantly increased only in slow‐5 (neither slow‐4 nor the typical range), which suggests abnormal network dynamics linked with very slow functional fluctuation. In a previous study, Luo and colleagues detected SZ‐related abnormalities of dFC strength in different frequency bands, which were associated with clinical symptoms of SZ patients in slow‐5 and slow‐4 (Y. L. Luo et al., 2020). Therefore, current work provides new insight into abnormal brain state dynamics in SZ in the frequency sub‐bands.

The current study has exhibited altered frequency‐dependent temporal dynamics in SZ, while their spatial patterns remained to be further studied. A recent CAP study reported that the SZ patients showed altered temporal dynamics, while the spatial maps were unaffected (Wang et al., 2021). Nevertheless, the consensus features identified in the following classification analysis might indicate different spatial patterns between SZ and HC to a certain degree. However, SZ patients might be characterized by more or fewer brain states, and future studies should consider performing the k‐means clustering within the patient group separately.

### 4.4 SZ classification based on different frequency bands

Machine learning approaches provide a powerful tool to identify neuroimaging predictors for brain function, behavioral phenotype as well as disorder‐related abnormality (Sui et al., 2020; Woo et al., 2017). Single‐subject prediction of SZ diagnosis is challenging but has the potential to guide psychiatry research and practice (A. Li et al., 2020; Reinen et al., 2018). Previous work found that the diagnostic status of SZ patients can be effectively predicted by using resting‐state dynamics of brain networks in the typical low‐frequency range (Kottaram et al., 2018). In the current study, we first evaluated the all‐patients model (69 SZ vs. 69 HC), and found that, combining CAP spatial features from slow‐4 and slow‐5 achieved the highest accuracy. The options for cross‐validation were considered, and we found that LOPO and LOO showed comparable classification results (Table S6), suggesting the impact of the cross‐validation method was minimal in this study. Our findings are consistent with previous studies that also reported that combining slow‐4 and slow‐5 helped improve the accuracy of patient classification (Huang et al., 2019; Tian et al., 2020).

However, the 69 HC subjects were also involved in the CAP definition, which might inflate the classification accuracy. Therefore, a hold‐out sample approach was performed to avoid potential bias. Rather than combining slow‐4 and slow‐5, slow‐4 achieved the highest accuracy of 0.8369, and slow‐5 achieved the lowest accuracy of 0.7583. Some other studies reported that compared with slow‐5, slow‐4 achieved higher accuracy (Hu et al., 2020) and provided more important features (Chen et al., 2016; Tian et al., 2020). Given that there were only a few studies focusing on the frequency‐specific aspects of functional brain networks and dynamics, the role of slow‐4 and slow‐5 for brain functioning is still unclear. Our results demonstrated that the methodological choice indeed impacted the classification results, in which a more rigorous choice (hold‐out sample approach) should be considered. To further our understanding, we asked if the unequal classification performance between slow‐4 and slow‐5 might be affected by the CAP definition of the hold‐out sample approach. To verify this assumption, we estimated the spatial similarity between the group‐averaged CAPs obtained by the 47 HC subjects and the individual CAPs of the remained 50 HC subjects. As shown in Figure S12, slow‐5 showed less spatial similarity (~0.7) than the typical range or slow‐4 (~0.8), suggesting the larger inter‐subject variabilities in slow‐5, and the less accurate projections from the group‐averaged CAPs to the novel individual could cause the lower classification accuracy. As for the features contributing to the classification, distinct consensus features from slow‐4 and slow‐5 were distributed in the FPN, DMN, and SMN (Figure 8). Dysfunction of these networks has often been implicated in psychotic neuropathology (Brandl et al., 2019; Sha et al., 2019). Furthermore, another independent dataset COBRE has also proved a certain degree of generalizability of the current study (Table S7). Although, the accuracy decreased to ~0.65–~0.7, it might be caused by the site differences or heterogeneity of SZ patients. In general, our results have shown the classification ability with CAP dynamics in different frequency bands.

### 4.5 Limitations

In this work, a priori sub‐bands were employed, including frequency divisions from slow‐5 to slow‐2, according to the literature (Buzsaki & Draguhn, 2004). The sub‐band definition might constrain the CAP results, although it has been widely used in previous fMRI studies (Han et al., 2011; Hou et al., 2014; Y. L. Luo et al., 2020; Zuo et al., 2010). Some other fMRI work utilized equally subdivided frequency bands (Ries et al., 2019), or a wavelet‐based method to obtain the frequency sub‐bands (F. F. Luo et al., 2020). Therefore, it is worth investigating the sub‐band properties of CAPs by using wavelet‐based or equally binned frequency bands in the future. Next, results based on slow‐3 and slow‐2 should be interpreted cautiously, because it is still under debate if higher‐frequency fMRI signals and network dynamics are influenced by noise arising from head motion, physiological signals or high‐frequency noise introduced in preprocessing (Agrawal et al., 2020; Laumann et al., 2017; Nalci et al., 2019; Trapp et al., 2018). Thus, the current study examined sub‐band CAPs in depth by focusing on slow‐5 and slow‐4 compared with the typical range. Finally, the resting‐state fMRI data used in the current study might be limited by the relatively short scan length of 8 min since previous literature recommended longer scan (Barber et al., 2021; Tomasi et al., 2017; Z. Yang et al., 2020). Future studies should take into account above mentioned methodological considerations and limitations for further evaluation.

## 5 CONCLUSIONS

The resting‐state CAP states have gradually varying spatial and temporal patterns across frequency sub‐bands in the healthy brain. Particularly, CAP states in slow‐4 exhibited stronger coactivation in several subcortical regions than slow‐5. As slow‐5 showed larger inter‐subject differences, slow‐4 achieved a better SZ prediction accuracy. Our work provides new evidence for functional dynamic brain and altered dynamic brain states in SZ from the perspective of multiple frequency bands, which can contribute to a better understanding of neuroimaging biomarkers for brain disorders.

## CONFLICT OF INTEREST

The authors declare no conflict of interest.

## AUTHOR CONTRIBUTIONS

**Hang Yang:** conceptualization, methodology, software, data analysis, writing original draft, editing, and revising. **Hong Zhang:** data analysis, and reviewing. **Chun Meng:** conceptualization, methodology, software, data analysis, editing, and revising. **Afra Wohlschläger:** reviewing and editing. **Felix Brandl:** reviewing and editing. **Xin Di:** reviewing and editing. **Shuai Wang:** reviewing and editing. **Lin Tian:** reviewing and editing. **Bharat Biswal:** conceptualization, methodology, software, editing, and revising.

## Supporting information


**Appendix S1** Supporting Information

## Data Availability

The WuXi data is not publicly available due to privacy or ethical restrictions. The Center for Biomedical Research Excellence (COBRE) dataset can be accessed at http://fcon_1000.projects.nitrc.org/indi/retro/cobre.html. The code that supports the findings of this study can be accessed at https://github.com/davidyoung1994/CoactivationPattern.
