# Supplementary material for: Frequency‐specific coactivation patterns in resting‐state and their alterations in schizophrenia: An fMRI study
Source: Hum Brain Mapp. 2022 Apr 27;43(12):3792–808. doi: 10.1002/hbm.25884 (PMC9294298; doi:10.1002/hbm.25884)
Supplement: Supplementary file 1 — Appendix S1 Supporting Information [file HBM-43-3792-s001.docx]

Supporting Information

**Data Preprocessing**

All structural and functional MRI images were preprocessed using DPABI (<http://rfmri.org/dpabi>). The T1-weighted images were first coregistered to the functional images, and then segmented into gray matter, white matter and cerebrospinal fluid by using DARTEL. For the resting-state fMRI images, the first 5 time points were removed to avoid instability of the scanner, and the remaining images were realigned to correct the head movement. Framewise displacement (FD) was calculated for each subject (Di & Biswal, 2015), and subjects with maximum translation or rotation FD greater than 2 mm or 2° were excluded from further analysis. The fMRI images were normalized to the Montreal Neurologic Institute (MNI) space using the deformation field maps obtained from the T1 segmentation, and resampled to 3 × 3 × 3 mm^3^. The mean white matter, cerebrospinal fluid and global signal, and 24 head motion parameters (Friston et al., 1996) were regressed from the time series. The time series was further detrended and temporal filtered. Besides the typical filtering bandpass (0.01–0.08 Hz), another four sub-bands including slow-5 (0.01–0.027 Hz), slow-4 (0.027–0.073 Hz), slow-3 (0.073–0.198 Hz) and slow-2 (0.198–0.25 Hz) were employed separately based on the previous study (Zuo et al., 2010). Finally, all images were smoothed using an 8 mm FWHM Gaussian kernel.

**Temporal Dynamics of CAP State**

In the manuscript, we have reported the results of **fraction of time**, **persistence** and **counts**. In addition to these state dominances that capture the inner-state dynamics, the **transition probability** between states was also calculated. **Resilience** is the probability or likelihood of remaining in the same state from one volume to the next volume, and it can be extracted from the diagonal of the transition probability matrix. Based on our previous research, resilience is highly related to persistence (Yang et al., 2021).

Considering the within-state transition probability (resilence) varied a lot from slow-2 (~ 0) to slow-5 (~ 0.8), as shown in Figure S3, the between-state transition probability also changed a lot (Figure S4), and statistical comparisons between sub-bands might not reveal the accurate frequency-specific effects on transition probability. Therefore, we only presented the results of transition probability in the supplementary materials.

**Validation in the COBRE Dataset**

To test the generalizability of our classification models, another independent dataset, the Center for Biomedical Research Excellence (COBRE) (http://fcon_1000.projects.nitrc.org/indi/retro/cobre.html) was used. After data quality check, 53 age- and gender-matched HC subjects (42 males, 11 females, age 18 - 65) and SZ patients (42 males, 11 females, age 18 - 65) were included.

A 3T Siemens Trio scanner was used to acquire the resting-state fMRI and T1-weighted structural MRI images. The resting fMRI data were collected with single-shot full k-space echo-planar imaging (EPI) with ramp sampling correction using the intercomissural line (AC-PC) as a reference, and the parameters were followed: TR = 2000 ms, TE = 29 ms, slice number = 33, slice thickness = 3.5 mm, flip angle = 75°, matrix size = 64 × 64, FOV = 240 mm, voxel size = 3.75 × 3.75 × 4.55 mm^3^, and volume number = 150. The parameters for the T1-weighted MPRAGE structural image were the following: TR = 2.53 s, TE = 1.64 ms, slice number = 192, slice thickness = 1 mm, matrix size = 256 × 256, FOV = 256 mm, and voxel size = 1 × 1 × 1 mm^3^.The same preprocessing pipeline was used for the COBRE dataset.

Firstly, we projected the CAP maps obtained from the 97 HC subjects from the WuXi dataset into each subject from the COBRE dataset. Then, the same level-one-pair-out classification pipeline (same with 69 HC vs 69 SZ) was performed. We found a lower classification accuracy for the COBRE dataset. This is not surprising as it might have been caused by the site differences or heterogeneity of schizophrenia patients. Nevertheless, the single-band results still achieved about 65% - 70% accuracy.

**The effects of filtering procedure on persistence**

A normalization strategy was performed to the persistence to evaluate the effects of filtering procedure. Specifically speaking, as described in (Buzsaki & Draguhn, 2004), the frequency sub-bands form a linear progression on a natural logarithmic scale (Example Figure 1). The center frequency of each band was listed in Table S8, and their corresponding periods are shown in Table S9. Then, the original persistence was divided by its period for each frequency band.

As shown in Figure S13, the normalized persistence across frequency bands was still unequal. Unlike the original persistence, the normalized persistence decreased from slow-2 to slow-5, suggesting the non-linear effects of the filtering procedure. A limitation is that the estimation of persistence was constrained by the sampling rate (TR = 2 s). For both slow-2 and slow-3, the averaged persistence is 2 seconds (one time point), which is the minimal persistence we can detect. Therefore, future studies should use shorter TR to study the dynamics at the higher frequency band (e.g., slow-2).

Besides, slow-4 and slow-5 showed similar persistence after normalization, hence a paired t-test was used to test the significance. The normalized persistence was still significantly different between slow-4 and slow-5 (p < 0.0001 for all the six CAPs). Future studies should also try to perform simulations to estimate the frequency-dependent dynamics.

**Statistics**

Instead of using two-way repeated ANOVA to estimate the group-frequency interactions and group main effects, simple effect analyses have also been performed for all the six CAP states. The temporal CAP differences between SZ and HC were compared in slow-5 and slow-4 separately, using a two-sample t-test with age and gender as covariates, and FDR correction was performed to account for the multiple comparisons.

As shown in Figure S10, SZ showed a consistently decreased fraction of time in the FPN-DMN-VN state (State 1 and 2), and an increased fraction of time in the SN-SMN-DMN state (State 3 and 4) in both slow-4 and slow-5. Moreover, SZ showed shorter persistence in the FPN-DMN-VN state (State 1 and 2) and FPN-DAN-DMN state (State 6) in slow4, while longer persistence in the SN-SMN-DMN state (State 3 and 4) was found in slow-5. As for the counts, SZ showed more counts in State 4 in slow-4, fewer counts in State 1 and 2 and more counts in State 3 in slow-5. No significant difference was found for transition probability in either slow-4 or slow-5 after FDR correction.

**References**

Buzsaki, G., & Draguhn, A. (2004). Neuronal oscillations in cortical networks. *Science, 304*(5679), 1926-1929. doi:10.1126/science.1099745

Di, X., & Biswal, B. B. (2015). Characterizations of resting-state modulatory interactions in the human brain. *J Neurophysiol, 114*(5), 2785-2796. doi:10.1152/jn.00893.2014

Friston, K. J., Williams, S., Howard, R., Frackowiak, R. S., & Turner, R. (1996). Movement-related effects in fMRI time-series. *Magn Reson Med, 35*(3), 346-355. doi:10.1002/mrm.1910350312

Yang, H., Zhang, H., Di, X., Wang, S., Meng, C., Tian, L., et al. (2021). Reproducible Coactivation Patterns of Functional Brain Networks Reveal the Aberrant Dynamic State Transition in Schizophrenia. *Neuroimage*, 118193. doi:10.1016/j.neuroimage.2021.118193

Zuo, X. N., Di Martino, A., Kelly, C., Shehzad, Z. E., Gee, D. G., Klein, D. F., et al. (2010). The oscillating brain: complex and reliable. *Neuroimage, 49*(2), 1432-1445. doi:10.1016/j.neuroimage.2009.09.037


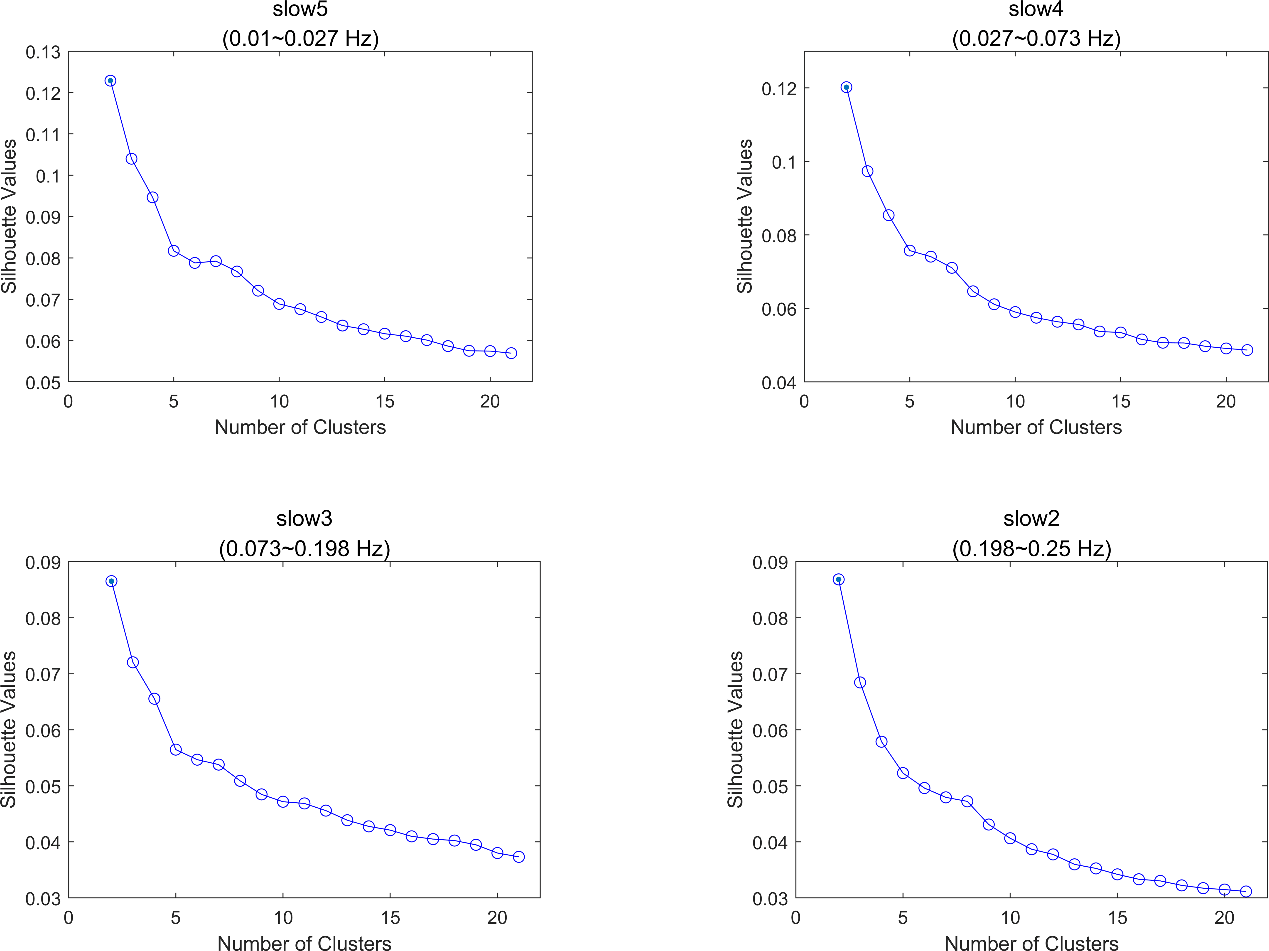


**Figure S1.** The clustering curves of the four sub-frequency bands. Silhouette value was calculated from k = 2 to k = 21 with step length = 1, and the silhouette values were monotonically decreasing with the increase of k. The elbow point for the four curves was around 5 to 7, and k = 6 was chosen in the manuscript.


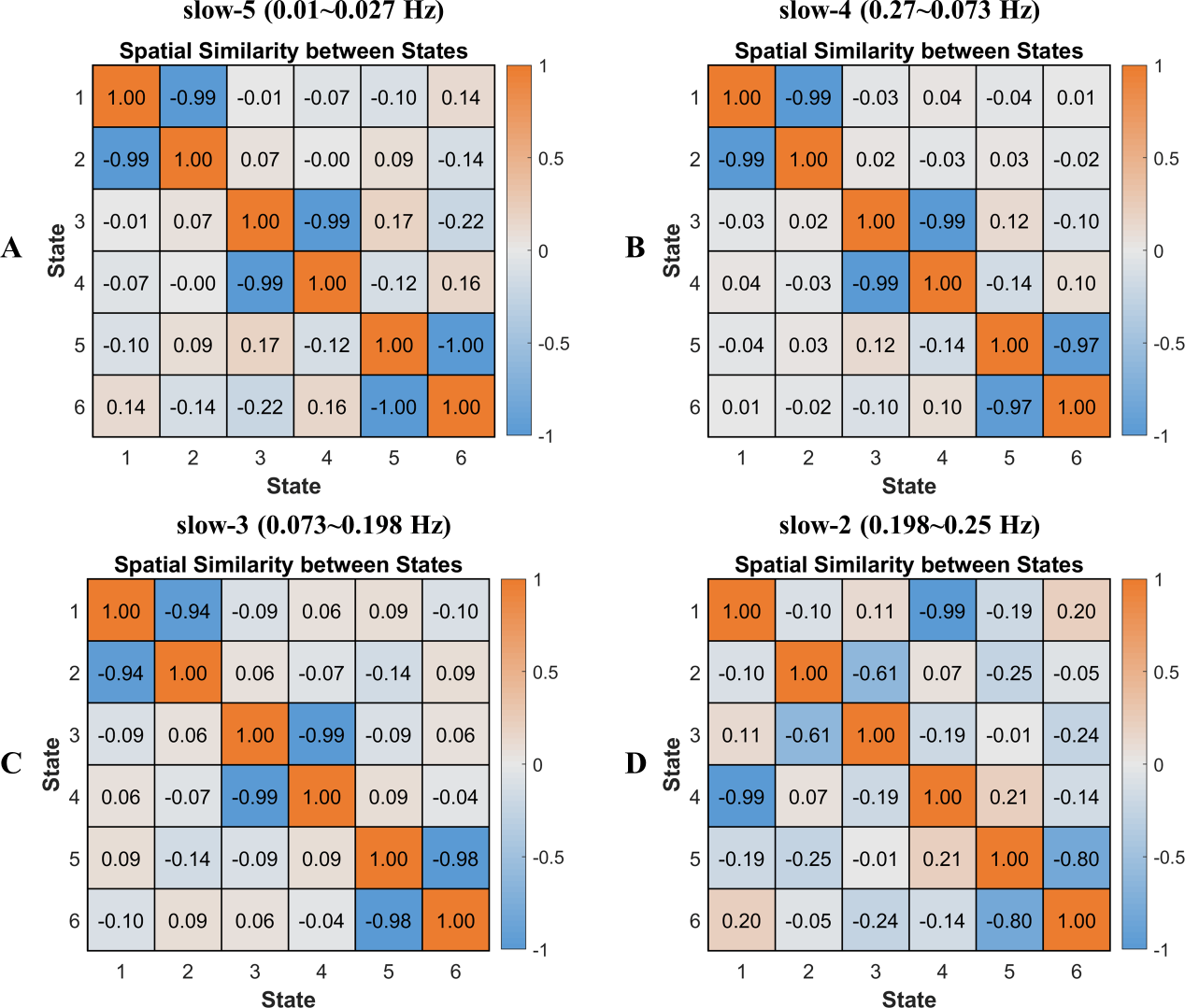


**Figure S2.** The CAP spatial similarity within (A) slow-5, (B) slow-4, (C) slow-3 and (D) slow-2. Pearson correlation was calculated to measure their spatial similarity. The colorbar shows the R-value.


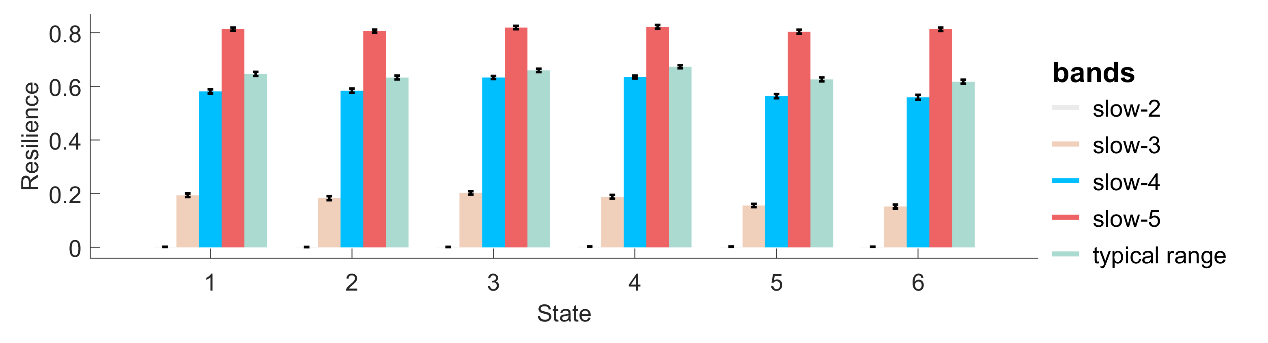


**Figure S3.** The resilience (within-state transition probability) of HC across slow-5 to slow-2.


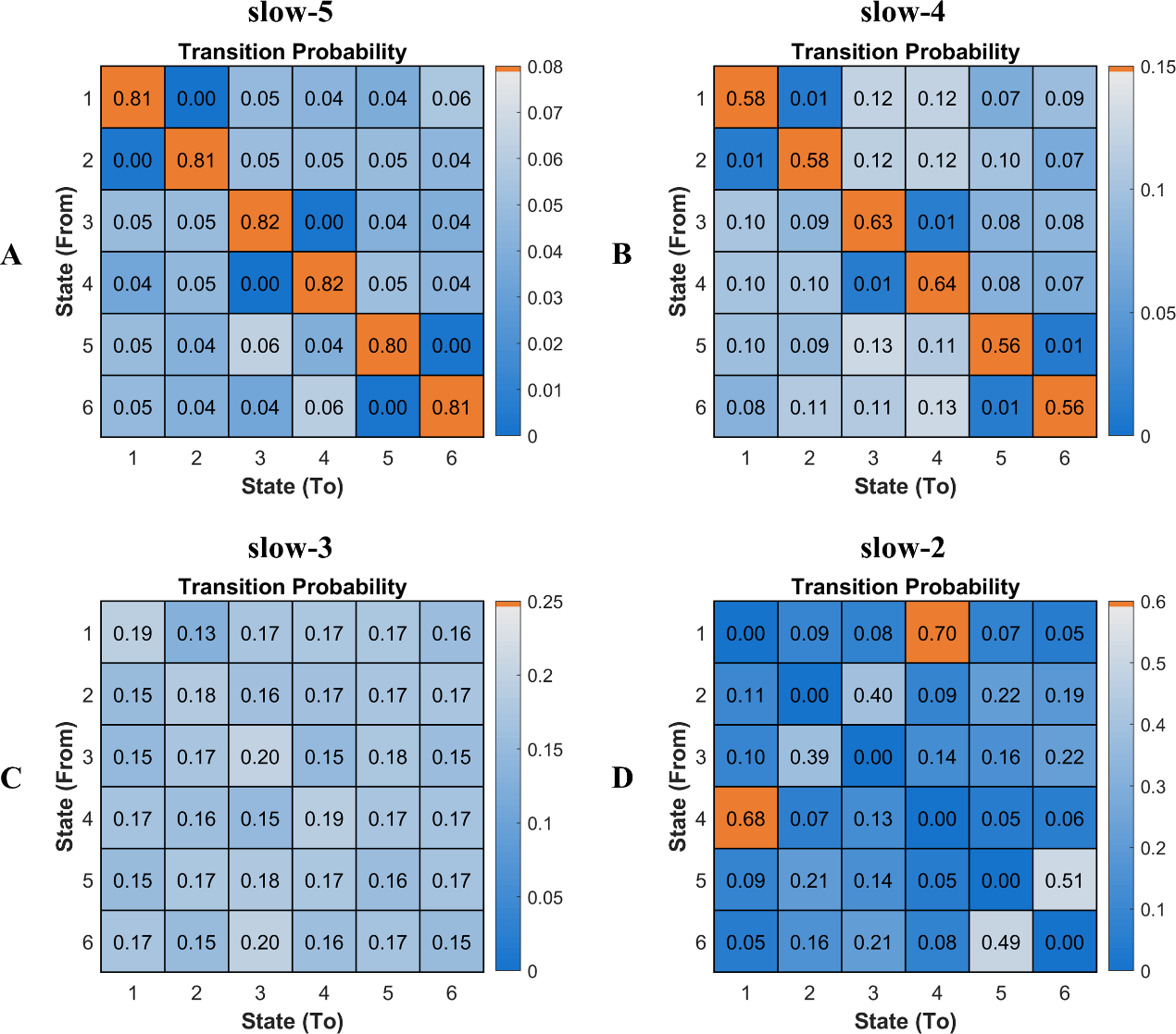


**Figure S4.** The average transition probability matrix within HC group from slow-5 to slow-2.


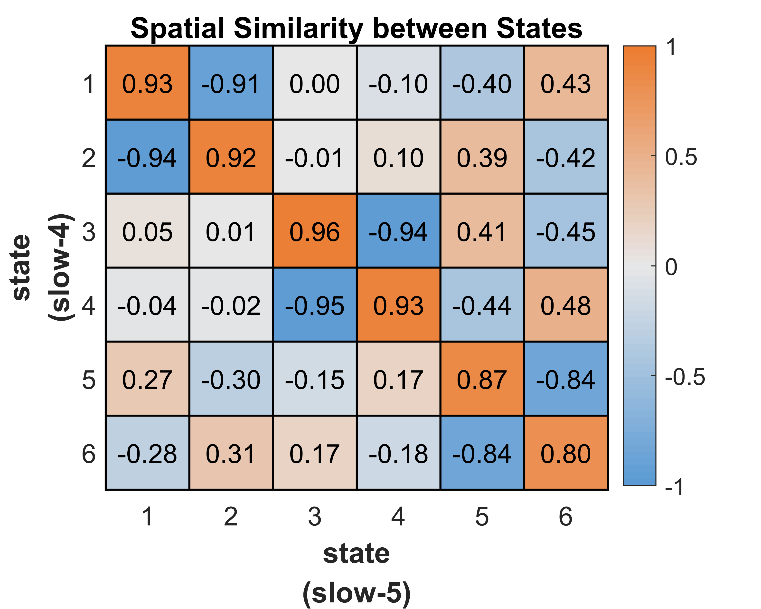


**Figure S5.** The CAP spatial similarity between slow-4 and slow-5. Pearson correlation was calculated to measure their spatial similarity. The colorbar shows the R-value.


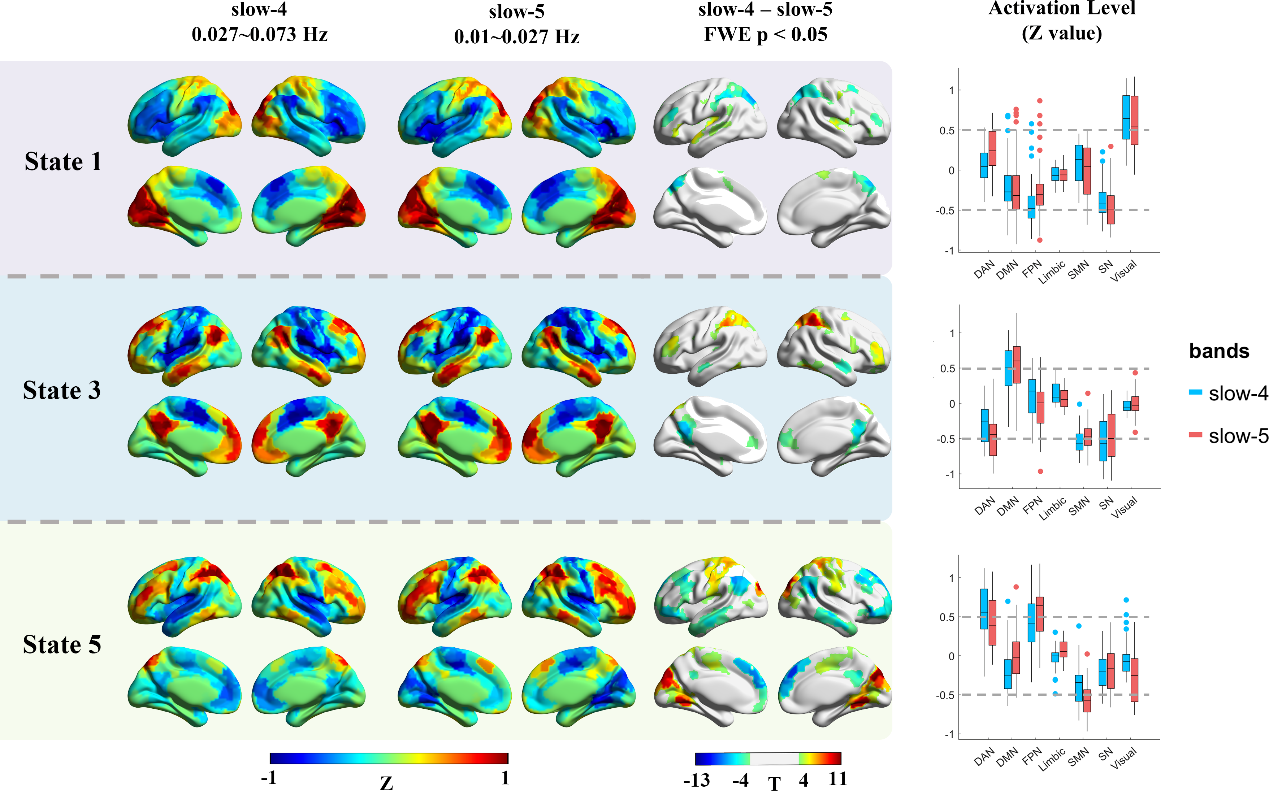


**Figure S6.** The frequency-specific effects between slow-4 and slow-5 within the HC group. The results of three states were presented, as the six CAP states were grouped into three pairs, and similar results were found within the pair. The first two columns show the cortical coactivations, and the color of each ROI indicates the activation deviation from its baseline level (Z-value). Paired t-test was performed for each state separately, and Bonferroni correction was used at the ROI level. The colorbar shows the T-value, and regions with P < 0.05 (FWE corrected) were presented in the third column. The last column shows the activation level of the seven networks in slow-4 and slow-5, and each point represents an ROI’s group averaged activation level from all 97 HC subjects.

**Abbreviations:** DAN, dorsal attention network; DMN, default mode network; FPN, fronto-parietal network; SN, salience network; SMN, somatomotor network.


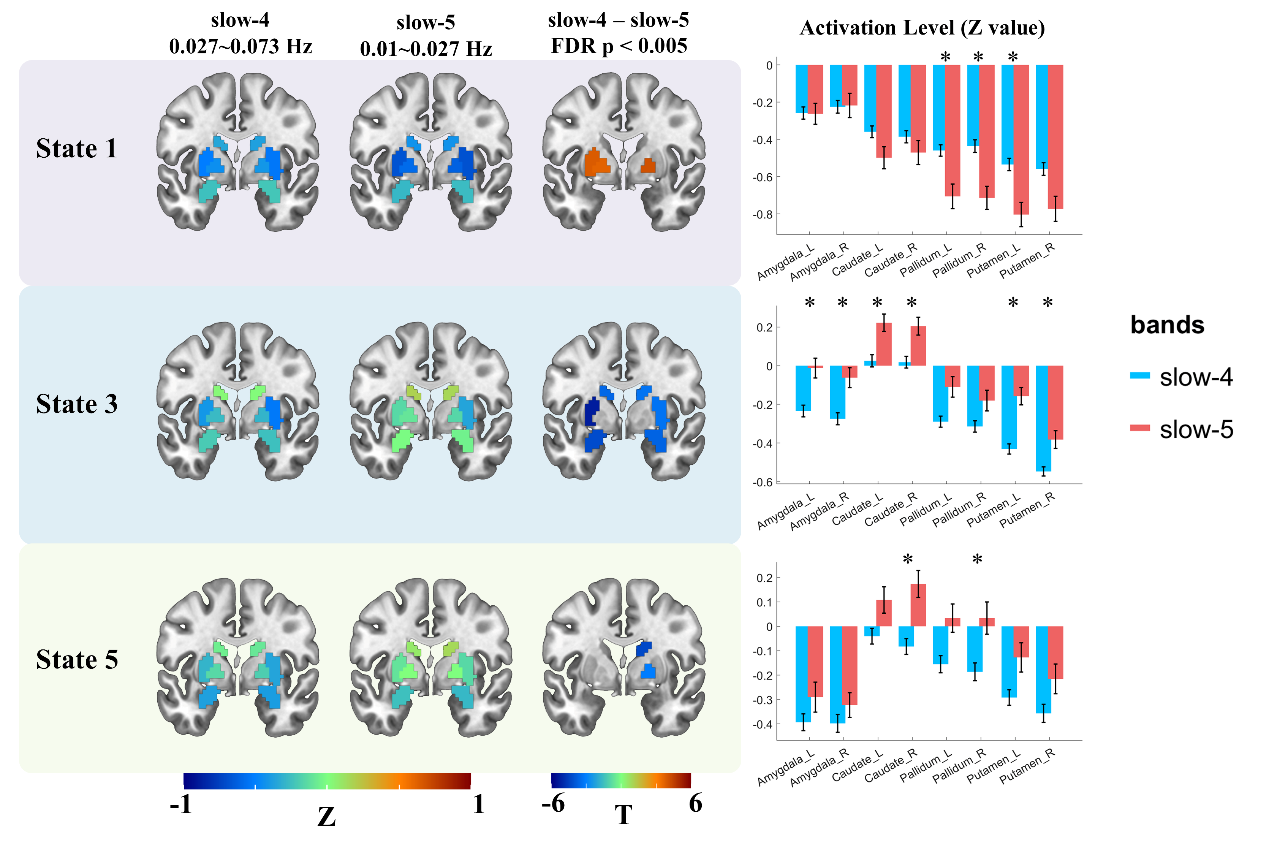


**Figure S7.** The subcortical activation differences between slow-4 and slow-5 within the HC group. The first two columns show the coactivation level of the eight subcortical regions, and the color of each ROI indicates the activation deviation from its baseline level (Z-value). Paired t-test was performed for six states separately, and FDR correction was used at the ROI level. Regions with P < 0.005 (FDR adjusted) were presented in the third row, and the colorbar shows the T-value. The last column shows the activation level of the eight subcortical regions in slow-4 and slow-5, from all 97 HC subjects.


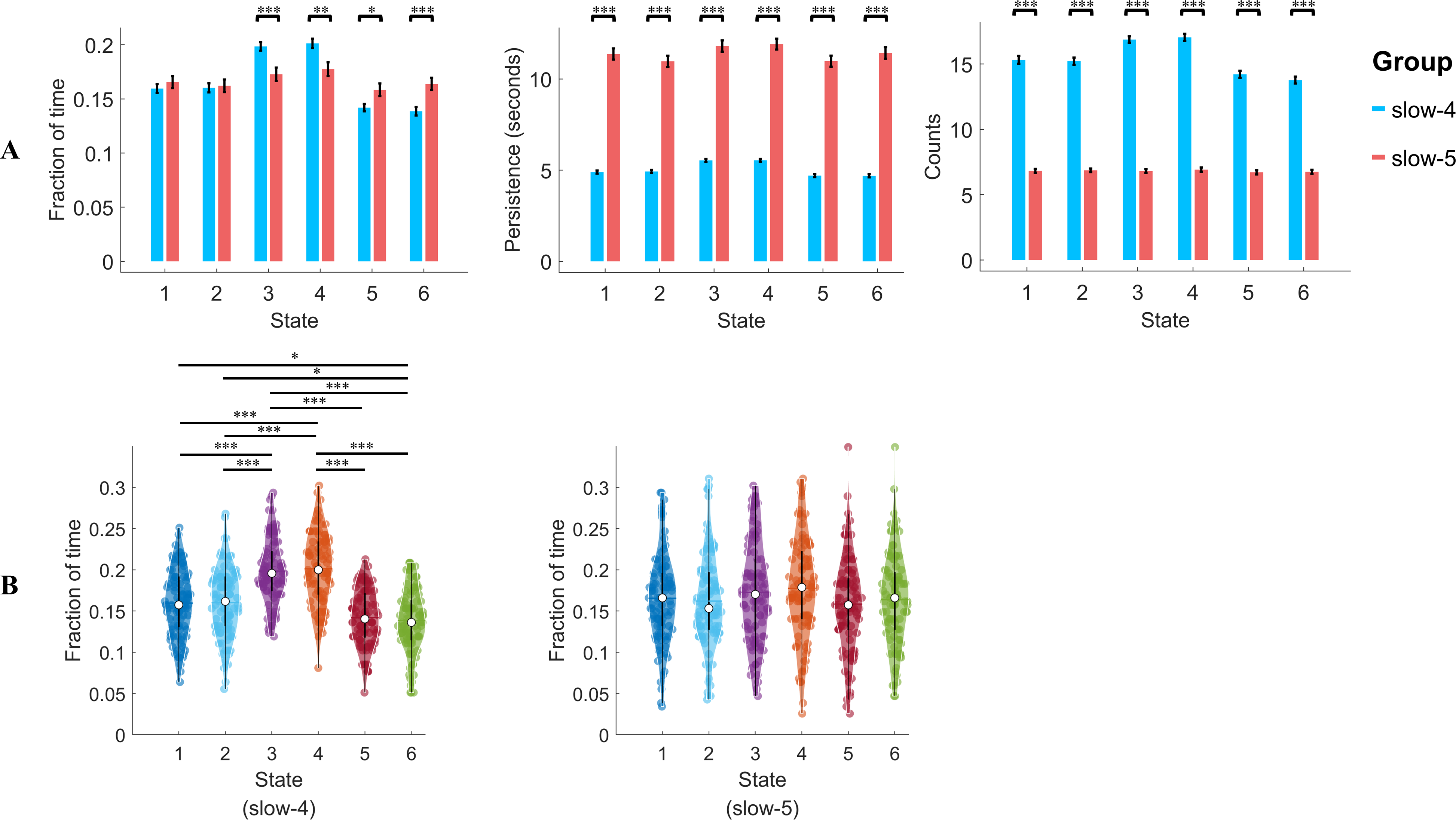


**Figure S8.** (A) The state temporal dominances (fraction of time, persistence, counts) under slow-4 and slow-5 within the HC group. (A) significant differences between slow-5 and slow-5 were detected by using paired t-test. (B) The fraction of time differed between six states in slow-4 but not in slow-5. Paired t-test was performed between each pair of states separately. Error-bar shows the standard error. * indicates p < 0.05, and ** indicates p < 0.005, and *** indicates p < 0.0005 separately, with FDR correction.


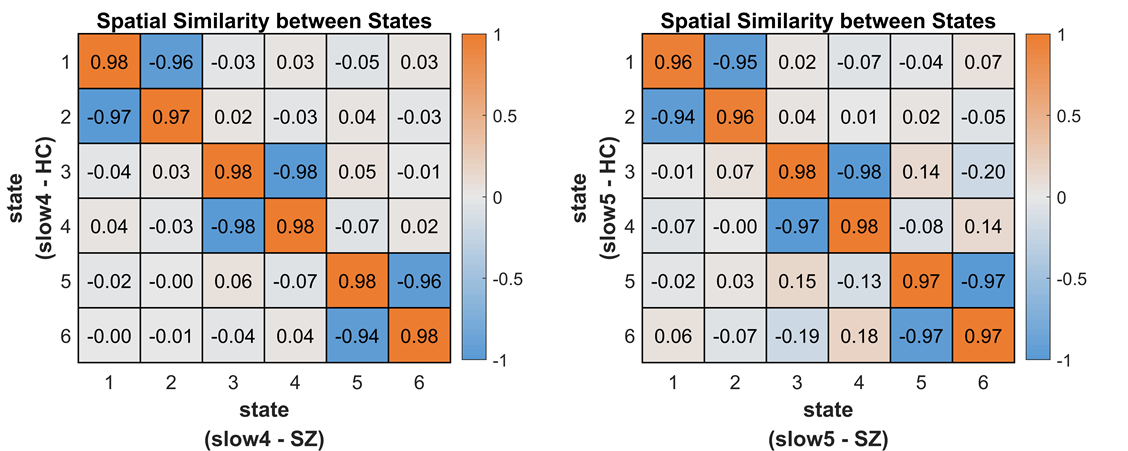


**Figure S9.** The CAP spatial similarity between SZ and HC in slow-4 and slow-5. Pearson correlation was calculated to measure their spatial similarity. The colorbar shows the R-value.


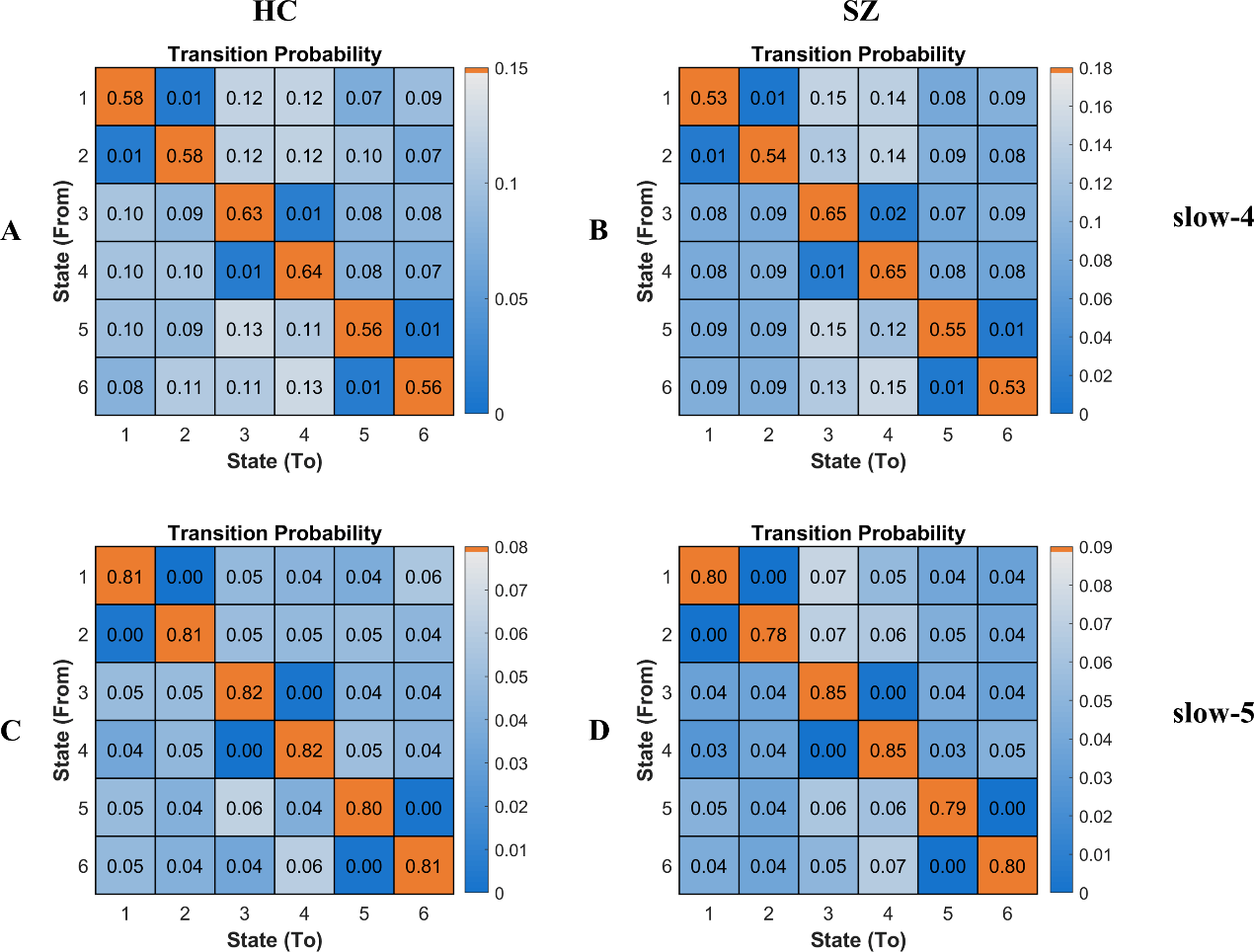


**Figure S10.** The transition probability matrix of HC and SZ in slow-4 and slow-5.


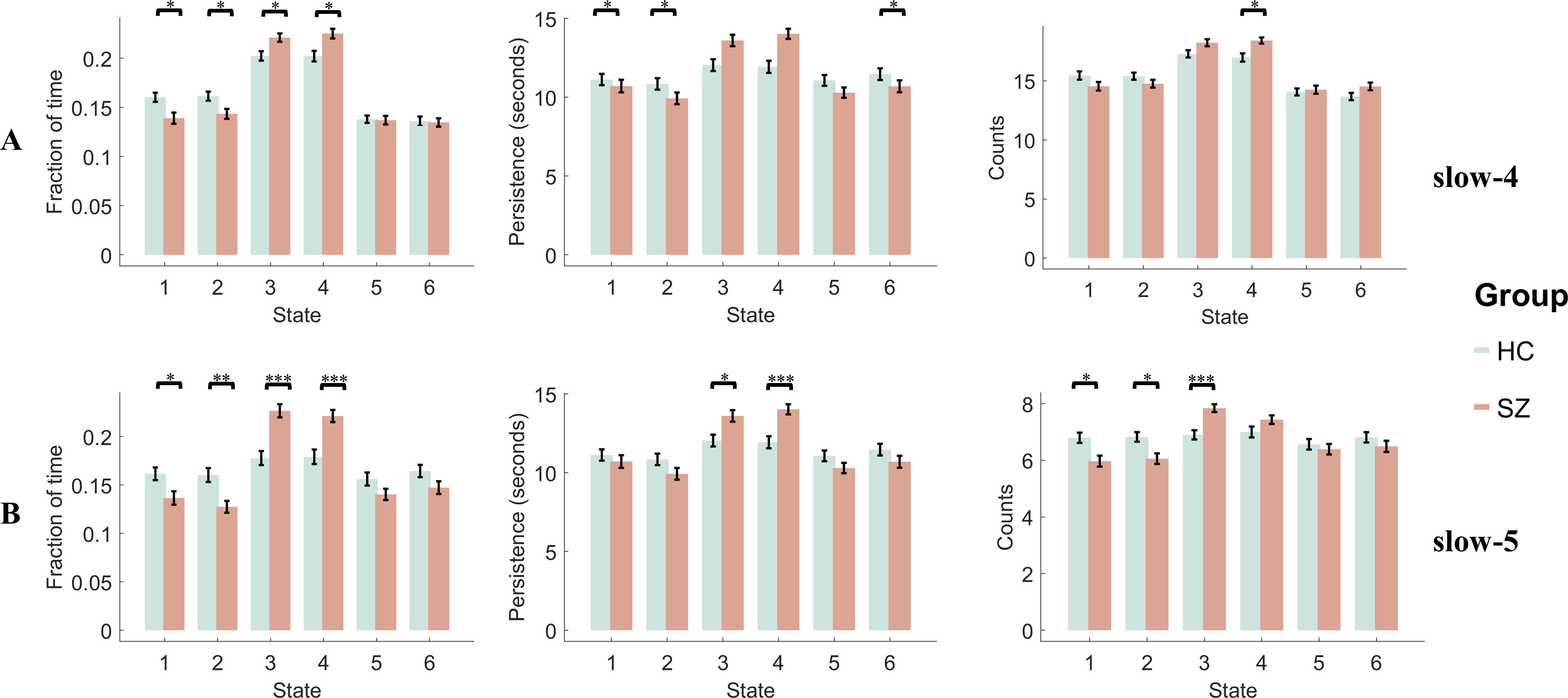


**Figure S11.** The state temporal dominances (fraction of time, persistence, and counts) under (A) slow-4 and (B) slow-5, and compared between SZ and HC using a two-sample t-test. Error-bar shows the standard error. * indicates p < 0.05, and ** indicates p < 0.005, and *** indicates p < 0.0005 separately, with FDR correction.


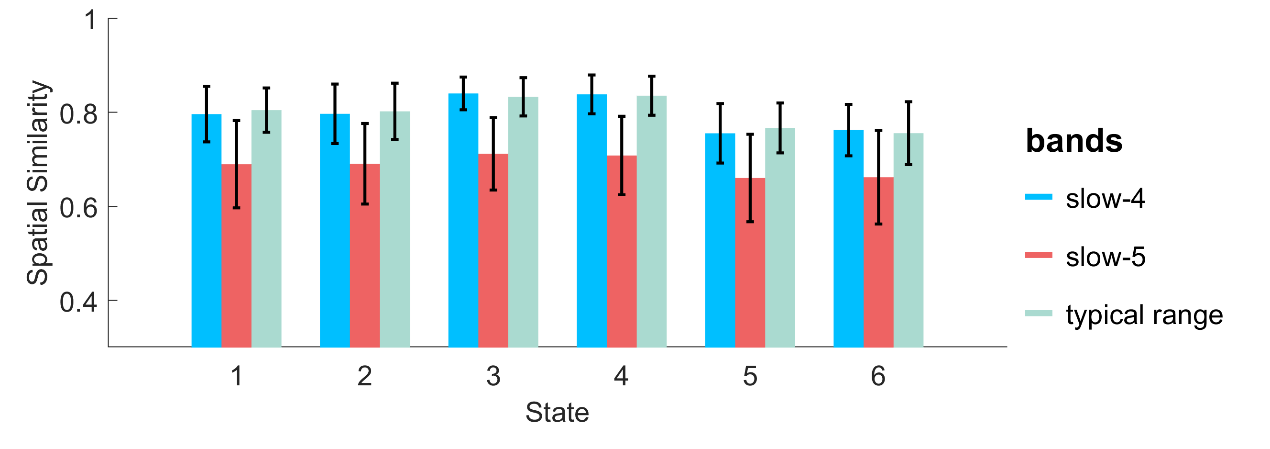


**Figure S12.** The spatial similarity between the group averaged CAPs of the CAP definition group and individual CAPs from the classification group. The spatial similarity was measured by using Pearson correlation.


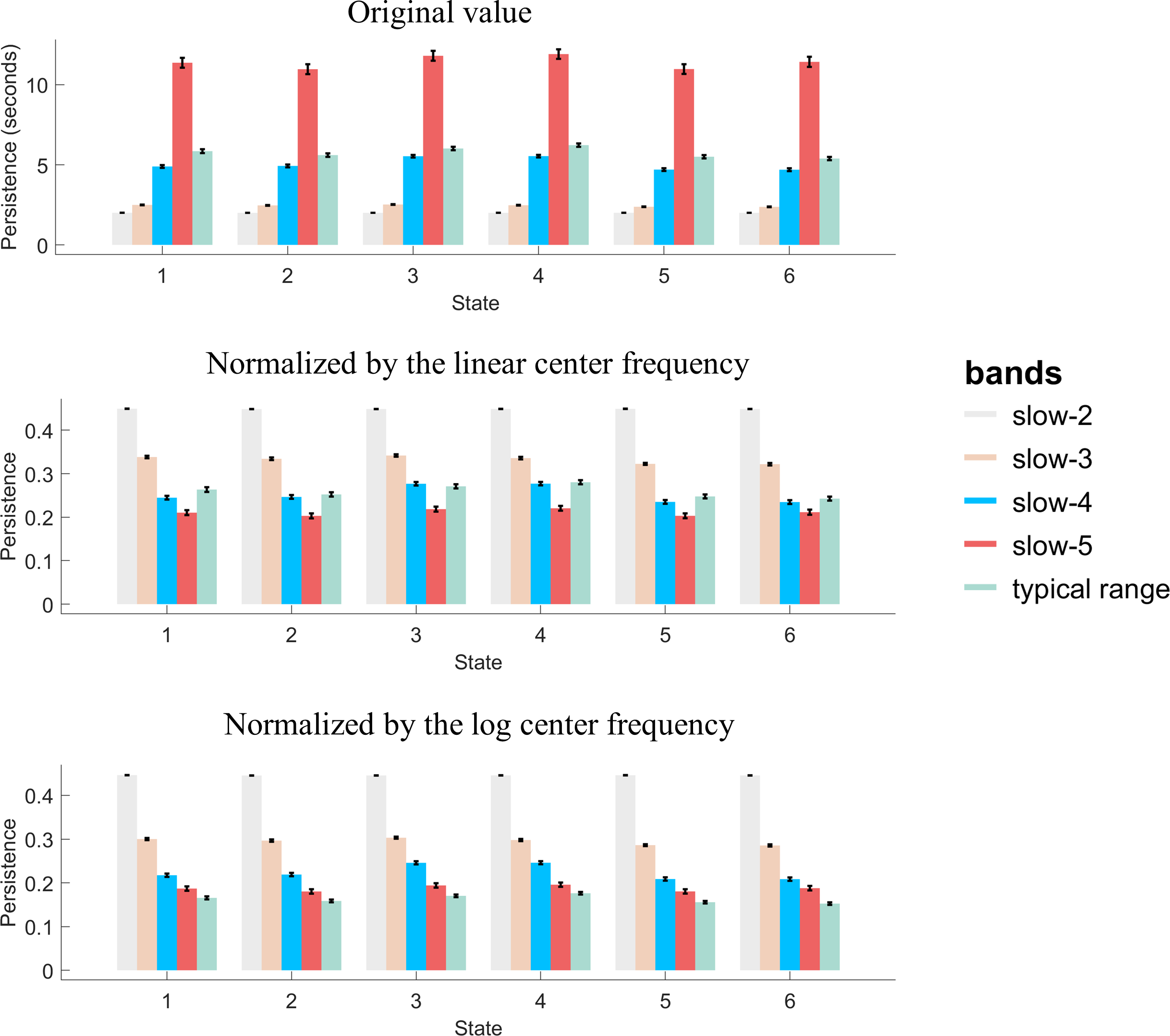


**Figure S13.** Normalized persistence based on the center frequency.

**Table S1.** The one-way repeated measures anova for CAP dynamics differences between different frequency bands in HC.

| **Fraction of time** | **F value** | **P value (FDR adjusted)** |
| --- | --- | --- |
| State 1 | 7.3688 | < 0.0001 |
| State 2 | 1.4190 | 0.2269 |
| State 3 | 15.3780 | < 0.0001 |
| State 4 | 13.7997 | < 0.0001 |
| State 5 | 6.8064 | < 0.0001 |
| State 6 | 14.4669 | < 0.0001 |
| **Persistence** | **F value** | **P value (FDR adjusted)** |
| State 1 | 675.4658 | < 0.0001 |
| State 2 | 629.3612 | < 0.0001 |
| State 3 | 733.0017 | < 0.0001 |
| State 4 | 800.4819 | < 0.0001 |
| State 5 | 622.3878 | < 0.0001 |
| State 6 | 635.6756 | < 0.0001 |
| **Counts** | **F value** | **P value (FDR adjusted)** |
| State 1 | 537.9231 | < 0.0001 |
| State 2 | 495.5708 | < 0.0001 |
| State 3 | 452.2484 | < 0.0001 |
| State 4 | 440.9961 | < 0.0001 |
| State 5 | 478.8121 | < 0.0001 |
| State 6 | 531.1890 | < 0.0001 |

**Table S2.** The paired t-test results for CAP dynamic differences between slow-4 and slow-5 in the HC group.

| **Fraction of time** | **T value** | **P value (FDR adjusted)** |
| --- | --- | --- |
| State 1 | -1.0788 | 0.3401 |
| State 2 | -0.3402 | 0.7345 |
| State 3 | 4.6502 | 0.0001^*^ |
| State 4 | 3.6518 | 0.0008^*^ |
| State 5 | -2.6540 | 0.0140^*^ |
| State 6 | -4.0468 | 0.0003^*^ |
| **Persistence** | **T value** | **P value (FDR adjusted)** |
| State 1 | -21.4037 | <0.0001^*^ |
| State 2 | -20.6626 | <0.0001^*^ |
| State 3 | -20.7937 | <0.0001^*^ |
| State 4 | -21.8191 | <0.0001^*^ |
| State 5 | -21.0289 | <0.0001^*^ |
| State 6 | -21.1770 | <0.0001^*^ |
| **Counts** | **T value** | **P value (FDR adjusted)** |
| State 1 | 28.5528 | <0.0001^*^ |
| State 2 | 30.6306 | <0.0001^*^ |
| State 3 | 41.5672 | <0.0001^*^ |
| State 4 | 32.7739 | <0.0001^*^ |
| State 5 | 26.2321 | <0.0001^*^ |
| State 6 | 22.1921 | <0.0001^*^ |

^*^ indicates the FDR-adjusted p-value < 0.05.

**Table S3.** The two-sample t-test results for CAP dynamics differences between SZ and HC in slow-5.

| **Fraction of time** | **T value** | **P value (FDR adjusted)** |
| --- | --- | --- |
| State 1 | -2.6171 | 0.0148^*^ |
| State 2 | -3.4805 | 0.0014^*^ |
| State 3 | 4.8993 | 1.6367e-05^*^ |
| State 4 | 4.2655 | 0.0001^*^ |
| State 5 | -1.7698 | 0.0790 |
| State 6 | -1.8570 | 0.0786 |
| **Persistence** | **T value** | **P value (FDR adjusted)** |
| State 1 | -0.7672 | 0.4443 |
| State 2 | -1.7484 | 0.1550 |
| State 3 | 2.9974 | 0.0097^*^ |
| State 4 | 4.1817 | 0.0003^*^ |
| State 5 | -1.6401 | 0.1550 |
| State 6 | -1.4422 | 0.1819 |
| **Counts** | **T value** | **P value (FDR adjusted)** |
| State 1 | -3.0981 | 0.0059^*^ |
| State 2 | -3.0284 | 0.0059^*^ |
| State 3 | 4.4068 | 0.0001^*^ |
| State 4 | 1.7656 | 0.1196 |
| State 5 | -0.6590 | 0.5110 |
| State 6 | 1.1828 | 0.2568 |

^*^ indicates the FDR-adjusted p-value < 0.05.

**Table S4.** The two-sample t-test results for CAP dynamic differences between SZ and HC in slow-4.

| **Fraction of time** | **T value** | **P value (FDR adjusted)** |
| --- | --- | --- |
| State 1 | -2.9029 | 0.0086^*^ |
| State 2 | -2.5984 | 0.0156^*^ |
| State 3 | 2.9302 | 0.0086^*^ |
| State 4 | 3.1496 | 0.0086^*^ |
| State 5 | -0.1705 | 0.8649 |
| State 6 | -0.2026 | 0.8649 |
| **Persistence** | **T value** | **P value (FDR adjusted)** |
| State 1 | -3.2723 | 0.0081^*^ |
| State 2 | -2.9275 | 0.0120^*^ |
| State 3 | 1.5616 | 0.1811 |
| State 4 | 1.2092 | 0.2745 |
| State 5 | -1.0259 | 0.3068 |
| State 6 | -2.3135 | 0.0444^*^ |
| **Counts** | **T value** | **P value (FDR adjusted)** |
| State 1 | -1.7997 | 0.1112 |
| State 2 | -1.4180 | 0.1902 |
| State 3 | 2.1838 | 0.0922 |
| State 4 | 3.3455 | 0.0064^*^ |
| State 5 | 0.4108 | 0.6819 |
| State 6 | 1.9082 | 0.1112 |

^*^ indicates the FDR-adjusted p-value < 0.05.

**Table S5.** The group (SZ and HC) and frequency (slow-4 and slow-5) main effects of CAP dynamics.

| **Fraction of time** | **F value** | **P value (FDR adjusted)** |
| --- | --- | --- |
| **Group** | | |
| State 1 | 10.89 | 0.0032 |
| State 2 | 14.82 | 8.17 × 10^-4^ |
| State 3 | 23.85 | 5.17 × 10^-5^ |
| State 4 | 21.76 | 6.56 × 10^-5^ |
| **Frequency** | | |
| State 4 | 6.85 | 0.0127 |
| State 5 | 5.02 | 0.0320 |
| State 6 | 16.77 | 9.97 × 10^-5^ |
| **Persistence** | **F value** | **P value (FDR adjusted)** |
| **Group** | | |
| State 2 | 5.46 | 0.0343 |
| State 3 | 9.99 | 0.0044 |
| State 4 | 17.96 | 2.48 × 10^-4^ |
| **Frequency** | | |
| State 1 | 586.68 | < 0.0001 |
| State 2 | 489.54 | < 0.0001 |
| State 3 | 798.34 | < 0.0001 |
| State 4 | 866.51 | < 0.0001 |
| State 5 | 712.15 | < 0.0001 |
| State 6 | 622.09 | < 0.0001 |
| **Counts** | **F value** | **P value (FDR adjusted)** |
| **Group** | | |
| State 1 | 8.30 | 0.0092 |
| State 2 | 6.59 | 0.0204 |
| State 3 | 13.51 | 0.0010 |
| State 4 | 13.79 | 0.0010 |
| **Frequency** | | |
| State 1 | 1.03 × 10^3^ | < 0.0001 |
| State 2 | 1.31 × 10^3^ | < 0.0001 |
| State 3 | 2.20 × 10^3^ | < 0.0001 |
| State 4 | 1.54 × 10^3^ | < 0.0001 |
| State 5 | 894.46 | < 0.0001 |
| State 6 | 810.49 | < 0.0001 |

**Table S6.** The classification results (69 SZ vs. 69 HC), WuXi dataset.

|  | **typical range** | **slow-5** | **slow-4** | **slow-5 + slow-4** |
| --- | --- | --- | --- | --- |
| **AUC** | 0.9443/0.9542 | 0.9200/0.9223 | 0.9477/0.9421 | 0.9630/0.9605 |
| **ACC** | 0.8841/0.9027 | 0.8551/0.8493 | 0.8841/0.8864 | 0.8913/0.9014 |
| **SE** | 0.8986/0.9193 | 0.8696/0.8687 | 0.8696/0.8825 | 0.8986/0.9139 |
| **SP** | 0.8696/0.8861 | 0.8406/0.8307 | 0.8986/0.8904 | 0.8841/0.8890 |

Note: The first value represents the classification results of leave-one-out, and the second value represents the classification results of level-one-pair-out.

Abbreviation: AUC, area under curve; ACC, accuracy; SE, sensitivity; SP, specificity.

**Table S7.** The classification results (53 SZ vs. 53 HC), COBRE dataset.

|  | **typical range** | **slow-5** | **slow-4** | **slow-5 + slow-4** |
| --- | --- | --- | --- | --- |
| **AUC** | 0.7430 | 0.6753 | 0.6717 | 0.6635 |
| **ACC** | 0.6953 | 0.6535 | 0.6562 | 0.6464 |
| **SE** | 0.7032 | 0.6592 | 0.6583 | 0.6391 |
| **SP** | 0.6874 | 0.6477 | 0.6542 | 0.6538 |

Abbreviation: AUC, area under curve; ACC, accuracy; SE, sensitivity; SP, specificity.

**Table S8.** Frequency band characteristics.

| Class | Linear Scale (Hz) | | | Natural Logarithmic Scale (lnHz) | | |
| --- | --- | --- | --- | --- | --- | --- |
|  | Low | Center | High | Low | Center | High |
| slow-2 | 0.198 | 0.224 | 0.250 | -1.62 | -1.50 | -1.39 |
| slow-3 | 0.073 | 0.136 | 0.198 | -2.62 | -2.12 | -1.62 |
| slow-4 | 0.027 | 0.050 | 0.073 | -3.62 | -3.12 | -2.62 |
| slow-5 | 0.010 | 0.019 | 0.027 | -4.62 | -4.12 | -3.62 |
| typical Range | 0.010 | 0.045 | 0.080 | -4.62 | -3.57 | -2.53 |

**Table S9.** The corresponding period of the center frequency.

| Period (s) | slow-2 | slow-3 | slow-4 | slow-5 | typical range |
| --- | --- | --- | --- | --- | --- |
| Linear | 4.46 | 7.38 | 20 | 54.05 | 22.22 |
| Natural Logarithmic | 4.49 | 8.32 | 22.52 | 60.86 | 35.36 |
